# Spp1 Contributes to Nano-Antimony Trioxide-Induced Male Reproductive Toxicity Associated with Inflammatory Response and Blood–Testis Barrier-Related Alterations

**DOI:** 10.3390/toxics14070569

**Published:** 2026-06-28

**Authors:** Zhenyao Huang, Yiwei Zhao, Yang Wang, Lei Jin, Jiali Yuan, Hao Meng, Jing Li

**Affiliations:** 1 Department of Respiratory and Critical Care Medicine, Second Affiliated Hospital of Xuzhou Medical University, Xuzhou 221004, China; huangzhenyao@xzhmu.edu.cn; 2 Key Laboratory of Human Genetics and Environmental Medicine, School of Public Health, Xuzhou Medical University, Xuzhou 221004, China; 304110120916@stu.xzhmu.edu.cn (Y.Z.); jiali.yuan@xzhmu.edu.cn (J.Y.); 3 First School of Clinical Medicine, Xuzhou Medical University, Xuzhou 221004, China; 202505020123@stu.xzhmu.edu.cn; 4 Medical Biological Experiment Credit Center, Basic Medical College, Xuzhou Medical University, Xuzhou 221004, China; jinlei@xzhmu.edu.cn; 5 Key Laboratory of Fire Protection and Retardant Technology, Ministry of Emergency Management, Chengdu 610036, China

**Keywords:** nano-antimony trioxide, reproductive toxicity, Spp1, blood–testis barrier, inflammatory response

## Abstract

Nano-antimony trioxide (Nano-Sb_2_O_3_) is extensively utilized in industrial production and consumer products, leading to widespread environmental contamination and human exposure. Accumulating evidence has demonstrated that Nano-Sb_2_O_3_ induces male reproductive toxicity, yet the underlying molecular mechanisms remain poorly understood. In this study, male C57BL/6 mice were exposed to Nano-Sb_2_O_3_ (2.5, 5.0, and 7.5 mg/kg/day). Exposure to Nano-Sb_2_O_3_ induced dose-dependent reproductive toxicity, evidenced by dose-dependent reductions in sperm motility (56.70% to 36.10%) and sperm density (15.76 × 10^6^/mL to 2.79 × 10^6^/mL) and a marked elevation in sperm malformation rates (4.56% to 44.36%), as well as severe histopathological alterations, testicular Sb accumulation, and elevated reactive oxygen species (ROS) levels. Transcriptomic analysis revealed significant enrichment of the PPAR and PI3K-Akt signaling pathways and identified SPP1 as one of the most significantly differentially expressed genes. Computational pathway perturbation analyses further yielded hypothesis-generating evidence supporting the potential involvement of PPAR signaling suppression and PI3K-Akt and inflammatory pathway activation following Nano-Sb_2_O_3_ exposure. Both mRNA and protein levels of SPP1 were significantly upregulated in a dose-dependent manner in mouse testes and TM4 Sertoli cells. In vitro experiments further demonstrated that Nano-Sb_2_O_3_ increased the expression of pro-inflammatory cytokines IL-1β and IL-6 by up to 5.6-fold and 4.7-fold, respectively, while impairing Sertoli cell viability and wound-healing capacity. Importantly, Spp1 silencing attenuated inflammatory responses and restored the expression of blood–testis barrier (BTB)-associated proteins, including ZO-1, Claudin-11, and N-cadherin. These findings suggest that SPP1 may contribute to Nano-Sb_2_O_3_-induced inflammatory responses and alterations in BTB-associated proteins, thereby potentially participating in male reproductive injury.

## 1. Introduction

Antimony trioxide (Sb_2_O_3_), a highly effective synergistic flame retardant, is widely used in industrial fields including electronics and electrical engineering, construction, and textiles [1]. China, the world’s major antimony-producing country, has experienced intensified regional environmental exposure risks due to antimony mining and smelting activities [2]. Although Sb_2_O_3_ contributes to fire safety in industrial and consumer applications, it is continuously released into the environment via waste gas, wastewater, and solid waste during its production, application, and disposal, resulting in soil, water, and air pollution, thereby posing potential threats to ecosystems and human health [3,4]. Field investigations from antimony mining areas demonstrated that the average antimony concentration in village rainwater runoff reached 128.6 μg/L, with the maximum concentration exceeding 450 μg/L, which is far above the national surface water standard limit of 5 μg/L [5]. Long-term rainfall erosion and atmospheric deposition further promote the migration and accumulation of antimony in water and soil, leading to persistent low-dose human exposure through respiratory inhalation, drinking water ingestion, and dermal contact. Sb_2_O_3_ has been listed as a priority pollutant by many countries, and its adverse health effects have attracted considerable attention [6].

However, the widespread adoption of nanomanufacturing has led to the release of substantial amounts of nano-sized antimony trioxide (Nano-Sb_2_O_3_) during production and application. Compared with bulk Sb_2_O_3_, Nano-Sb_2_O_3_ features smaller particle size, larger specific surface area and superior biological penetrability. Its small particle size and large specific surface area may facilitate translocation across biological barriers following inhalation exposure, thereby increasing its potential to reach distant organs and induce enhanced toxic effects.

Accumulated evidence demonstrates that nano-antimony trioxide (Nano-Sb_2_O_3_) can be absorbed by humans and mammals via respiratory inhalation, oral ingestion and dermal contact, exerting toxicity in multiple organs [7]. Nano-Sb_2_O_3_ exposure induces respiratory injuries such as airway inflammation, pneumonitis and chronic antimony pneumoconiosis, as well as cardiovascular dysfunction including QT prolongation, arrhythmia and myocardial damage [8]. It also causes prominent hepatotoxicity and nephrotoxicity, characterized by liver cell injury, acute renal failure and renal tubular dysfunction [9,10]. Moreover, Nano-Sb_2_O_3_ triggers neurological impairments such as peripheral neuropathy, cerebellar ataxia and ototoxicity, accompanied by hematological abnormalities including hemolytic anemia and cytopenia, as well as typical skin lesions and contact dermatitis [11,12]. Antimony trioxide has been classified as a Group 2B possible human carcinogen by the International Agency for Research on Cancer (IARC), with animal and epidemiological evidence suggesting its potential tumorigenic risk. Nevertheless, current studies mainly focus on its respiratory, cardiovascular, hepatic, renal, neurological and carcinogenic toxicities. The research on male reproductive toxicity induced by Nano-Sb_2_O_3_ exposure remains severely insufficient. To date, only gross phenotypic alterations, including impaired sperm quality and testicular histological damage have been reported, while the underlying molecular regulatory mechanisms, candidate signaling pathways, and regulatory genes involved in Nano-Sb_2_O_3_-induced reproductive toxicity remain largely unexplored [13].

Recent studies have demonstrated that environmental contaminants can impair male reproductive function through inflammatory activation, oxidative stress, and disruption of testicular homeostasis. Chromium-containing compounds have been reported to induce testicular injury and spermatogenic dysfunction, whereas microplastic exposure has been associated with blood–testis barrier impairment, inflammatory responses, and reduced sperm quality. These findings suggest that the male reproductive system is particularly vulnerable to emerging environmental pollutants and underscore the importance of investigating the reproductive toxicity of Nano-Sb_2_O_3_ [14,15].

Among the various targets of environmental toxicants, the blood–testis barrier (BTB) has attracted considerable attention because of its critical role in maintaining the spermatogenic microenvironment. Composed primarily of tight junctions and intercellular junctions between Sertoli cells, the BTB is essential for normal spermatogenesis, and its disruption represents a key event in male infertility [16,17]. Inflammatory response is recognized as a critical mediator of BTB impairment [18]. Numerous studies have demonstrated that environmental pollutants such as bisphenol A, phthalates, cadmium, lead, microplastics and perfluorinated compounds, can trigger inflammatory responses, alter the expression of BTB-associated proteins, and contribute to impairment of BTB function, and eventually result in male reproductive toxicity. As the major cellular component of the BTB, testicular Sertoli cells (TM4) can release inflammatory factors under inflammatory stimulation, thereby potentially impairing BTB function [19,20].

Based on these observations, we hypothesized that inflammatory responses and alterations in BTB-associated proteins may contribute to Nano-Sb_2_O_3_-induced male reproductive toxicity. Therefore, this study aimed to identify candidate molecular pathways and regulatory genes involved in Nano-Sb_2_O_3_-induced reproductive injury using integrated in vivo and in vitro approaches.

## 2. Materials and Methods

### 2.1. Experimental Animals and Chemicals

Nano antimony trioxide (Nano-Sb_2_O_3_, particle size: 50 nm, purity ≥ 99.9%, batch number: 20240905) was purchased from Beijing DK Nano Technology Co., Ltd. and diluted with normal saline to prepare the stock solution.

Six-week-old C57BL/6 mice were obtained from the Laboratory Animal Center of Xuzhou Medical University. All mice were acclimated for 10 days under specific pathogen-free (SPF) conditions before the formal experiment. Mice were then randomly divided into four groups with five mice in each group: three Nano-Sb_2_O_3_-exposed groups and one control group (C, normal saline). The mice in the Nano-Sb_2_O_3_-exposed groups were treated with Nano-Sb_2_O_3_ via intranasal instillation for 16 consecutive days at doses of 2.5 mg/kg/d (low dose group), 5 mg/kg/d (medium dose group), and 7.5 mg/kg/d (high dose group) [21,22], respectively. The control group received an equal volume of normal saline. The exposure doses of Nano-Sb_2_O_3_ (2.5, 5.0, and 7.5 mg/kg/day) were selected with reference to the National Toxicology Program (NTP) inhalation studies of antimony trioxide, which employed multiple graded exposure concentrations to evaluate toxicological effects. Considering the different exposure route used in the present study, three dose levels were established to represent low-, medium-, and high-dose exposure groups and to assess the dose-dependent reproductive toxicity of Nano-Sb_2_O_3_ [23]. After 16 days of treatment, the testes, epididymides, and sperm were collected for subsequent analysis. All animal studies were approved by the Institutional Animal Care and Use Committee of Xuzhou Medical University (IACUC No. 202312T043, Approval Date: 14 December 2023). At the end of exposure, mice were anesthetized with sodium pentobarbital and euthanized by cervical dislocation prior to tissue collection.

### 2.2. Characterization of Nano-Sb_2_O_3_ Particles

The morphology of Nano-Sb_2_O_3_ was observed using a field emission scanning electron microscope (FE SEM, Zeiss, Oberkochen, Germany). Briefly, Nano-Sb_2_O_3_ powder was dispersed in ethanol at a concentration of 1 mg/mL by ultrasonication, dropped onto a silicon wafer, and dried at room temperature before observation. The hydrodynamic size distribution and zeta potential of Nano-Sb_2_O_3_ in aqueous suspension were determined by dynamic light scattering (DLS) using a particle size analyzer (Bettersize, Dandong, China). For DLS measurement, Nano-Sb_2_O_3_ was dispersed in ultrapure water at a concentration of 0.5 mg/mL to prepare a working suspension, followed by ultrasonication for 10 min to ensure uniform dispersion before analysis. The specific surface area and pore characteristics of Nano-Sb_2_O_3_ were determined by nitrogen adsorption–desorption isotherms using the Brunauer–Emmett–Teller (BET) method. Approximately 0.1 g of sample was degassed at 120 °C for 6 h prior to measurement. Nitrogen adsorption was performed at −196 °C, and the specific surface area was calculated from the adsorption isotherm. Total pore volume and average pore diameter were derived from the desorption branch using the Barrett–Joyner–Halenda (BJH) method. All measurements were conducted in triplicate.

### 2.3. In Vivo Animal Experiments

#### 2.3.1. Determination of Antimony Concentration in Testicular Tissues

Testicular tissues were collected, weighed, and digested with 65% HNO_3_ and 30% H_2_O_2_ using a microwave digestion system (LabTech, Beijing, China) at 180 °C for 25 min. The digest was diluted to 3.0 mL with ultrapure water. Sb concentrations were determined by ICP-OES (5110, Agilent Technologies, Santa Clara, CA, USA) at Sb 206.833 nm with Y as internal standard. The calibration curve ranged from 10 to 1000 μg/L (R^2^ > 0.999). Spike recovery experiments yielded 94–107%. Sb content was expressed as μg/g wet tissue weight.

#### 2.3.2. Determination of Testicular Organ Coefficient and Cross-Sectional Measurement

Prior to the experiment, the body weight of each mouse was measured and recorded. Subsequently, the testis weight of each mouse was measured separately to calculate the organ coefficient, which was calculated using the following formula: Organ coefficient (%) = (Testis weight/Mouse body weight) × 100%. The cross-sectional area of testis was calculated by measuring the transverse diameter with a ruler, and the measured data were recorded for quantitative analysis [24]. All measurement methods were performed in accordance with standardized protocols to ensure the accuracy and reproducibility of the experimental results.

#### 2.3.3. Preparation and Quality Assessment of Sperm Suspension

After euthanasia, bilateral epididymides were isolated from mice, thoroughly rinsed with warm PBS, and transferred to an EP tube containing 1 mL of normal saline pre-warmed to 37 °C. The epididymides were rapidly minced with ophthalmic scissors, gently pipetted to form a uniform sperm suspension, and used for subsequent quality assessments [25].

For sperm abnormality rate analysis, 10 μL of the sperm suspension was smeared on a slide and observed under an optical microscope. Sperm abnormalities were defined as hookless, banana-shaped, large-headed, amorphous, tail-folded, double-headed, or double-tailed. A total of 500 spermatozoa were counted in different fields of view per slide to calculate the sperm abnormality rate [26].

For sperm motility rate assessment, 10 μL of the sperm suspension was observed under an optical microscope, and 200 spermatozoa were counted. Sperm exhibiting non-progressive movement (e.g., circling in place), slow progressive movement (e.g., slow forward or rotational movement), medium-speed linear forward movement, or fast linear forward movement were classified as motile. The sperm motility rate was calculated as the ratio of motile sperm number to the total counted sperm number [27].

For sperm density determination, 10 μL of the sperm suspension was loaded onto a hemocytometer and counted under an optical microscope. The total number of sperm in five squares within the central large grid was recorded as A, and the sperm density (per mL) was calculated using the formula: A × 5 × 10^4^ [28].

#### 2.3.4. Observation of Testicular Tissue Damage by HE Staining

Frozen testicular tissues were taken, rewarmed at 4 °C, and then transferred to 4% paraformaldehyde for gradient rewarming and fixation for 24 h. Subsequently, the tissues were subjected to dehydration, paraffin embedding, sectioning (5 μm thick), and HE staining (hematoxylin staining for nuclei for 3–5 min, eosin staining for cytoplasm for 1–2 min, tap water bluing, gradient ethanol dehydration, xylene clearing, neutral gum mounting) [29]. The morphology of seminiferous tubules and cell arrangement (such as spermatogenic cell shedding and interstitial edema) were observed under an optical microscope, photographed, and recorded.

#### 2.3.5. Measurement of Reactive Oxygen Species (ROS)

Reactive oxygen species (ROS) levels in testicular tissues were determined using a commercial ROS assay kit based on 2′,7′-dichlorodihydrofluorescein diacetate (DCFH-DA) according to the manufacturer’s instructions. Briefly, testicular tissues were homogenized in ice-cold phosphate-buffered saline (PBS) and centrifuged at 12,000× *g* for 10 min at 4 °C. The resulting supernatants were incubated with DCFH-DA working solution at 37 °C for 30 min in the dark. Fluorescence intensity was measured using a fluorescence microplate reader (Leica Microsystems, Wetzlar, Germany) at excitation and emission wavelengths of 488 and 525 nm, respectively. ROS levels were normalized to the control group and expressed as relative ROS levels.

### 2.4. Transcriptome Sequencing

Total RNA was extracted from testicular tissues using the TRIzol method, and its concentration, purity, and integrity were detected using a NanoDrop 2000 (Thermo Fisher Scientific, Wilmington, DE, USA) and an Agilent 2100 Bioanalyzer (RIN ≥ 7.0) (Agilent Technologies, Santa Clara, CA, USA). A total of 1 μg of qualified RNA was used, and sequencing libraries were constructed using the NEBNext Ultra RNA Library Prep Kit for Illumina: mRNA was enriched with oligo (dT) magnetic beads and then fragmented. Double-stranded cDNA was synthesized using random hexamers, followed by end repair, A-tailing, adapter ligation, and PCR amplification (15 cycles). The libraries were validated using an Agilent 2100 detection. Qualified libraries were sequenced on the Illumina NovaSeq X Plus platform with PE150 sequencing (Illumina, San Diego, CA, USA), and the sequencing depth of each sample was ≥50 million reads. After quality control of the raw data using FastQC (v0.12.1) and removal of adapters and low-quality sequences (Q < 20) using Trimmomatic (v0.40), the clean data were aligned to the mouse reference genome GRCm39 using HISAT2. Gene expression was quantified as FPKM using HTSeq (v2.0.5), and differentially expressed genes (DEGs) were analyzed using DESeq2 with the criteria of |log_2_FC| ≥ 1 and padj < 0.05. Functional enrichment analysis of DEGs was performed using the KEGG and GO databases.

### 2.5. Computational Pathway Perturbation Analysis

To computationally support the potential involvement of PPAR and PI3K-Akt signaling, three orthogonal in silico analyses were performed using differentially expressed genes (DEGs, |log_2_FC| ≥ 1, *p* < 0.05) identified from Nano-Sb_2_O_3_-treated versus control mouse testes. (1) LINCS L1000CDS^2^ connectivity mapping: Mouse DEGs were mapped to human orthologs and queried against the NIH LINCS L1000 database (https://maayanlab.cloud/L1000CDS2/) (accessed on 17 June 2026) in reverse mode to identify small molecules that produce gene expression signatures opposing the Nano-Sb_2_O_3_ DEG profile. (2) Enrichr pathway enrichment analysis: DEGs were analyzed using the Enrichr platform (https://maayanlab.cloud/Enrichr/) (accessed on 17 June 2026) against the KEGG 2021 Human, WikiPathways 2021 Human, and MSigDB Hallmark 2020 gene set libraries. Enrichment significance was assessed by Fisher’s exact test with *p* < 0.05 as the significance threshold. (3) PROGENy pathway activity inference: Based on the core responsive gene sets of 14 pathways defined by the PROGENy algorithm, the mean log_2_FC between treatment and control groups was calculated from the full transcriptome to infer pathway activity. Positive values (Score > 0.5) indicate pathway activation, and negative values (Score < −0.5) indicate pathway inhibition. Detailed results are provided in Supplementary Figure S5 and Supplementary Table S3.

### 2.6. Exploratory Bioinformatics Analysis of SPP1-Associated Pathways

To explore potential SPP1-associated pathways, we performed Spearman correlation analysis between SPP1 and selected genes (PPAR targets, PI3K-Akt components, inflammatory mediators, and BTB-associated proteins) using transcriptomic data from all six samples (*n* = 6; 3 treated + 3 control). Protein interaction networks were explored using STRING (v12.0), and upstream transcription factors were predicted using Enrichr (ChEA 2022 database; Ma’ayan Lab, New York, NY, USA). Given the limited sample size (*n* = 6) and the exploratory nature of these analyses, all findings were interpreted as hypothesis-generating and do not establish causality.

### 2.7. In Vitro Cellular Experiments

#### 2.7.1. Culture of TM4 Testicular Sertoli Cells

TM4 cells were resuscitated and inoculated into DMEM/F12 medium containing 10% FBS and 1% penicillin/streptomycin solution, then cultured in an incubator at 37 °C with 5% CO_2_. When the confluency reached 80–90%, the cells were digested with trypsin (0.25% trypsin at 37 °C for 2–3 min), and passaged at a ratio of 1:3 to prepare logarithmic phase cells for subsequent experiments [30].

#### 2.7.2. Cell Viability Assay (CCK8)

TM4 cells in logarithmic growth phase were seeded into 96-well plates. After complete adherence, cells were exposed to Nano-Sb_2_O_3_ solutions at gradient concentrations of 0, 1, 5, 10, 20, 40, 80, 160, 200 μmol/L (the 0 μmol/L group served as the normal control). Blank control and normal control groups were set up simultaneously. After incubation for 24 h, CCK-8 working solution was added to each well and incubated away from light for 2 h. The absorbance value at 450 nm was measured with a microplate reader, and cell viability was calculated accordingly.

#### 2.7.3. Cell Scratch Assay

Logarithmic-phase TM4 cells were seeded into 6-well plates at a density of 2 × 10^5^ cells/well. When cells reached 100% confluence, a scratch was made using a sterile pipette tip, and detached cells were washed twice with PBS. Immediately after scratching, cells were exposed to Nano-Sb_2_O_3_ at concentrations screened by CCK-8 assay (0, 5, 10, 20 μmol/L), with the 0 μmol/L group serving as the control (regular culture medium). Photographs of the scratch area were captured under an inverted microscope (Leica Microsystems, Wetzlar, Germany) at 0 h and 24 h. The scratch area was measured using ImageJ software (version 1.54f), and the cell migration rate was calculated according to the formula: Cell migration rate = [(Area at 0 h − Area at 24 h)/Area at 0 h] × 100% [31]. The effect of Nano-Sb_2_O_3_ on the migration function of TM4 cells was analyzed.

#### 2.7.4. SPP1 siRNA Transfection Assay

Logarithmic-phase TM4 cells were seeded and cultured until they reached appropriate confluence. The specific siRNA targeting Spp1 and negative control siRNA were transfected into cells using transfection reagent in strict accordance with the manufacturer’s instructions. After transfection, the original medium was replaced with fresh complete medium. Subsequent Nano-Sb_2_O_3_ exposure treatment and related functional experiments were performed after successful transfection. The transfection efficiency was verified by qRT-PCR before formal experiments.

#### 2.7.5. Western Blot

Phenylmethanesulfonyl fluoride (Beyotime Biotechnology, Shanghai, China) and RIPA Lysis Buffer (Beyotime Biotechnology, China) were used to extract the protein from the testes. The suspensions were sonicated and kept on ice for 30 min. The supernatants were collected after centrifuging the lysates at 13,000 *g* for 30 min at 4 °C. Protein concentrations were measured using the BCA Protein Quantification Kit (Vazyme, China). The protein samples were separated on sodium dodecyl sulfate-polyacrylamide gel electrophoresis and transferred to poly-vinylidene fluoride (PVDF) membranes. The PVDF membranes were blocked in 5% experimental grade non-fat milk or BSA for 1–2 h at RT and then incubated in primary antibodies overnight at 4 °C. The next day, the PVDF membranes were immediately incubated in the secondary antibodies at RT for 2 h after three washes with Tris-Buffered saline containing Tween buffer. The SuperSignal West Femto Chemiluminescent Substrate (Tanon, China) was used to visualize the signal of target proteins. Antibody information is detailed in Table S1 (Supporting Information).

#### 2.7.6. Quantitative Real-Time Polymerase Chain Reaction (qRT-PCR)

Total RNA was extracted from cells and tissues using TRIzol reagent Vazyme BioTech (Nanjing, China), following the manufacturer’s provided method. The total RNA was then quantified using the nanodrop 2000c system (Thermo Fisher Scientific, USA). The RNA was then reverse transcribed into cDNA using HiScript II Q RT SuperMix for qPCR (+gDNA wiper) (Vazyme BioTech (China), and amplified with ChamQ SYBR qPCR Master Mix (Applied Biosystems, Waltham, MA, USA) at a volume of 10 μL. Real-time fluorescence quantitative PCR was carried out based on the ChamQ SYBR qPCR Master Mix (Vazyme BioTech, China). The data were normalized using GAPDH. Primer information is detailed in Table S2 (Supporting Information) [32].

#### 2.7.7. Immunofluorescence Staining (IF)

TM4 cells were seeded onto sterile coverslips in 24-well plates and cultured until the confluency reached 60–70%. After treatment with the corresponding concentration of Nano-Sb_2_O_3_, the cells were washed three times with PBS, then fixed with pre-cooled (−20 °C) methanol for 15 min at room temperature. After three washes with PBS, the cells were incubated with blocking solution at room temperature. Subsequently, the cells were incubated with primary antibody (ZO 1, diluted 1:400) overnight at 4 °C. The next day, the cells were washed three times with PBST, then incubated with fluorescent secondary antibody at room temperature for 1 h in the dark. After three washes with PBST, the cell nuclei were stained with DAPI at room temperature for 5 min [30]. Finally, the coverslips were mounted with anti-fade mounting medium, and images were captured under a fluorescence microscope (Leica Microsystems, Wetzlar, Germany). Antibody information is detailed in Table S1 (Supporting Information).

#### 2.7.8. Apoptosis Analysis by Annexin V/PI Flow Cytometry

TM4 cells were treated with 20 μmol/L Nano-Sb_2_O_3_ for 48 h. Apoptosis was assessed by flow cytometry using FITC-Annexin V/PI double staining (BD FACSCanto II, Becton, Dickinson and Company, Franklin Lakes, NJ, USA). Data from three independent experiments are expressed as mean ± SD. Statistical significance was determined by unpaired two-tailed Student’s *t*-test (*p* < 0.05).

### 2.8. Statistical Analyses

All data are presented as mean ± standard deviation (SD). Statistical analysis was performed using GraphPad Prism software (version 9.0; GraphPad, San Diego, CA, USA). Prior to parametric analyses, normality was assessed using the Shapiro–Wilk test and homogeneity of variance was evaluated using the Brown–Forsythe test. All datasets satisfied the assumptions for parametric analysis. Comparisons among multiple groups were performed using one-way analysis of variance (ANOVA) followed by Dunnett’s multiple comparisons test. Comparisons between two groups were conducted using Student’s *t*-test. A value of *p* < 0.05 was considered statistically significant. For transcriptomic analyses, differential gene expression was performed using DESeq2. Multiple-testing correction was conducted using the Benjamini–Hochberg procedure, and genes with an adjusted *p* value (false discovery rate, FDR) < 0.05 were considered statistically significant.

## 3. Results

### 3.1. Characterization of Nano-Sb_2_O_3_ Particles

Nano-Sb_2_O_3_ was characterized using FE-SEM and DLS. FE-SEM images were captured at 20.00 K× magnification with a scale bar of 500 nm, and representative images from three independent visual fields are presented. DLS measurements were performed in triplicate. The hydrodynamic particle size distribution showed a peak at 297 nm with a polydispersity index (PDI) of 0.22. The zeta potential of the aqueous suspension was −13.6 ± 0.61 mV (Figure S1).

The nitrogen adsorption–desorption BET analysis revealed that the specific surface area was 18.5 m^2^ g^−1^, total pore volume was 0.086 cm^3^ g^−1^, and the average pore diameter was 18.6 nm, indicating mesoporous structure formed by particle stacking. The relative pressure at monolayer adsorption was 0.12.

### 3.2. Nano-Sb_2_O_3_ Accumulation Induces Oxidative Stress, Testicular Injury, and Sperm Quality Deterioration in Mice

**Figure 1:**
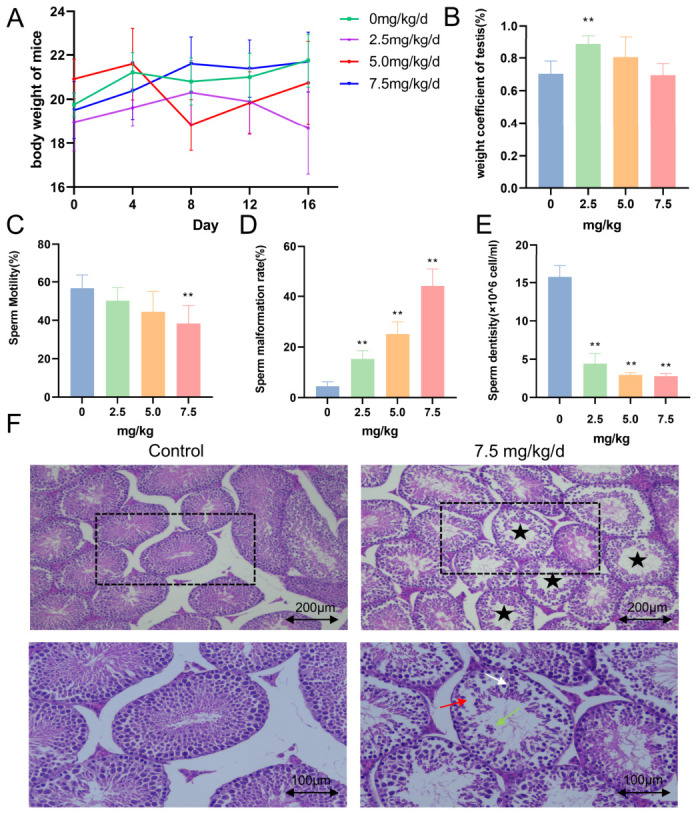
Effects of Nano-Sb_2_O_3_ exposure on male mice. (**A**) Body weight change during exposure. (**B**) Testicular organ coefficient. (**C**) Sperm motility. (**D**) Sperm malformation rate. (**E**) Sperm density. (**F**) H&E staining of testicular tissue (scale bars: 200 μm, 100 μm). Data are presented as mean ± SD (*n* = 5 per group). For Figure (**A**), body weight at each independent time point was analyzed separately using one-way analysis of variance (ANOVA). For Figures (**B**–**E**), one-way ANOVA was applied to compare indicators among different groups. All ANOVA analyses were followed by Dunnett’s multiple comparisons test. ** *p* < 0.01 versus the control group. Graphs were generated using GraphPad Prism 9.0.

Body weight showed similar trends across all groups with no significant differences (Figure 1A). The mean final body weights were 21.56 ± 1.16 g (0 mg/kg/d), 20.66 ± 1.08 g (2.5 mg/kg/d), 20.76 ± 1.71 g (5.0 mg/kg/d), and 20.78 ± 1.77 g (7.5 mg/kg/d), with no significant differences between groups (all *p* > 0.05). The testicular organ coefficient was significantly increased in the 2.5 mg/kg group (0.72 ± 0.09%, *p* < 0.05), slightly increased in the 5.0 mg/kg group (0.77 ± 0.07%, *p* > 0.05), and decreased in the 7.5 mg/kg group (0.69 ± 0.07%, *p* > 0.05), compared with the control group (0.70 ± 0.08%) (Figure 1B). Gross observation showed that testes in the high-dose group were pale and soft, with a significantly reduced testicular coefficient relative to the 2.5 mg/kg group (*p* < 0.05). There was no significant difference in testicular cross-sectional area among all groups (Figure S4A,B).

Sperm motility declined progressively with increasing Nano-Sb_2_O_3_ dosage, and was significantly decreased in the 7.5 mg/kg group (*p* < 0.01) (Figure 1C). The sperm motility rates were 56.70 ± 7.17% (control), 50.20 ± 7.11% (2.5 mg/kg/d), 48.40 ± 11.15% (5.0 mg/kg/d), and 36.10 ± 10.06% (7.5 mg/kg/d), with the 7.5 mg/kg group showing a significant reduction (*p* < 0.01 vs. control). The sperm malformation rate was significantly increased in each treatment group in a dose-dependent manner (*p* < 0.01) (Figure 1D). The rates were 4.56 ± 1.94% (control), 15.67 ± 3.43% (2.5 mg/kg/d), 25.13 ± 4.70% (5.0 mg/kg/d), and 44.36 ± 7.61% (7.5 mg/kg/d), with all treatment groups significantly higher than the control (all *p* < 0.01). Sperm density was significantly reduced in all dose groups and decreased with increasing exposure dosage (*p* < 0.01) (Figure 1E). The densities were 15.76 ± 1.53 × 10^6^/mL (control), 4.42 ± 1.33 × 10^6^/mL (2.5 mg/kg/d), 2.94 ± 0.31 × 10^6^/mL (5.0 mg/kg/d), and 2.79 ± 0.34 × 10^6^/mL (7.5 mg/kg/d), with all treatment groups significantly lower than the control (all *p* < 0.01).

HE staining revealed progressive histopathological lesions in mouse testes in a dose-dependent manner. In the control group, seminiferous tubules were intact and neatly arranged, with neatly layered spermatogenic cells and sufficient mature sperm inside the tubules, and normal interstitial tissue structure. In the high-dose group, seminiferous tubules were damaged, spermatogenic epithelium became thin and shed, the number of mature spermatozoa decreased markedly, spermatogenic cells were reduced in number and disorganized, amorphous substances appeared in the tubule cavity. Partial seminiferous tubules retained only Sertoli cells, and testicular interstitium showed loose edema (Figure 1F).

### 3.3. Transcriptome Analysis Reveals PPAR and PI3K-Akt Pathways

**Figure 2:**
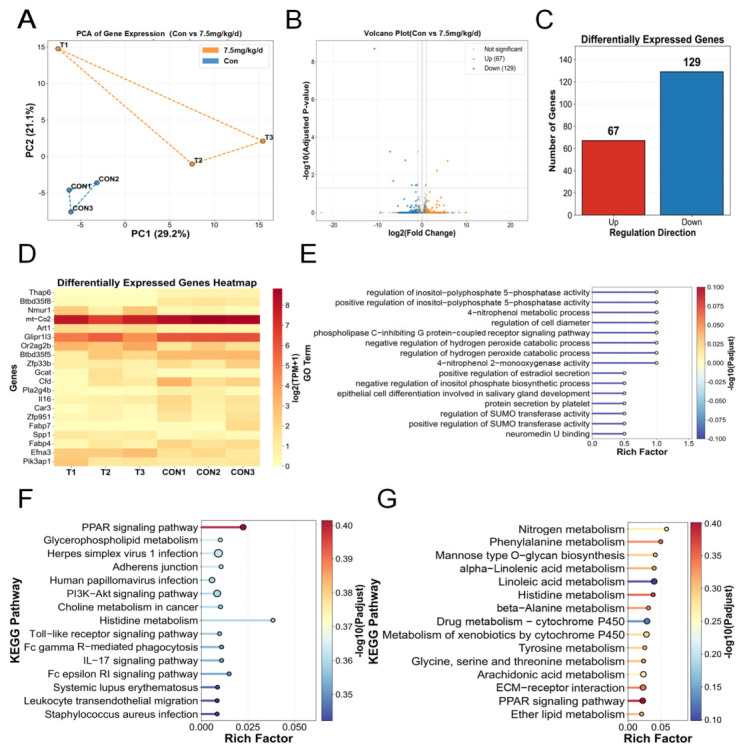
Transcriptomic analysis of mouse testes after Nano-Sb_2_O_3_ exposure. (**A**) Heatmap of differentially expressed genes (DEGs) (7.5 mg/kg/d vs. control). (**B**) Number of upregulated (67) and downregulated (129) DEGs. (**C**) Volcano plot of DEGs. (**D**) KEGG pathway enrichment analysis. (**E**) GO functional enrichment analysis. (**F**) Bubble plot of enriched suppressed KEGG pathways. (**G**) Bubble plot of enriched activated KEGG pathways (*n* = 3).

To explore the molecular mechanism of Nano-Sb_2_O_3_-induced testicular injury, we first examined Sb accumulation and oxidative stress in testicular tissues. Testicular Sb concentrations were significantly elevated in Nano-Sb_2_O_3_-exposed mice in a dose-dependent manner (Figure S2). In parallel, ROS levels in testicular tissues were significantly increased and exhibited a dose-dependent trend (Figure S3). Subsequently, transcriptome sequencing was conducted on testicular tissues of control and high-dose groups. PCA analysis revealed distinct separation of gene expression profiles between the two groups (Figure 2A). A total of 196 differentially expressed genes were screened, containing 67 upregulated and 129 downregulated genes (Figure 2B,C). The heatmap presents different gene expression characteristics among samples (Figure 2D).

GO enrichment analysis indicated that these DEGs were mainly involved in spermatogenesis, inflammatory response, cell adhesion and oxidative stress regulation, with some enrichment in metabolism-related biological processes (Figure 2E). KEGG pathway enrichment analysis of differentially expressed genes was performed and displayed by ranking on adjusted *p*-value significance and Rich Factor, respectively (Figure 2F,G). Ranking by significance revealed the most prominent enrichment in the PPAR signaling pathway, accompanied by significant enrichment of multiple signaling and immune-inflammatory pathways, including the PI3K-Akt signaling pathway. When ranked by Rich Factor, the enriched pathways were mainly involved in substance metabolism and drug metabolism, such as nitrogen metabolism, phenylalanine metabolism, linoleic acid metabolism, and cytochrome P450-mediated drug metabolism, indicating a higher proportion of differentially expressed genes in metabolic pathways. The PPAR signaling pathway was significantly enriched under both ranking criteria, suggesting that this pathway is stably and critically regulated in the biological effects induced by nano-Sb_2_O_3_ exposure.

### 3.4. Computational Analyses Support the Potential Involvement of PPAR, PI3K-Akt, and SPP1

To computationally support the potential involvement of PPAR and PI3K-Akt pathways in Nano- Sb_2_O_3_-induced male reproductive toxicity, we performed three orthogonal in silico analyses. First, LINCS L1000CDS^2^ connectivity mapping in reverse mode identified 50 small molecules that produce gene expression signatures opposite to the Nano-Sb_2_O_3_ DEG profile (top score: L-690,488, score = 0.161), including HDAC inhibitors (vorinostat, trichostatin A), CDK inhibitors (AT-7519), and NF-κB inhibitors (IMD 0354), suggesting that pharmacological perturbation of related signaling pathways can transcriptionally counteract the toxic effects of Nano-Sb_2_O_3_. Second, Enrichr multi-database pathway enrichment analysis confirmed significant enrichment of the PPAR signaling pathway in both KEGG and WikiPathways databases (KEGG FDR-adjusted *p* = 0.0148, Z-score = 11.50; WikiPathways *p* = 0.0122, Z-score = 12.75), with PI3K-Akt signaling showing borderline enrichment (*p* = 0.0586, core DEGs: EFNA3, SPP1, PIK3AP1). MSigDB Hallmark analysis further revealed TNF-α/NF-κB signaling and Inflammatory Response as the top two enriched pathways. Third, PROGENy pathway activity inference provided directional evidence demonstrating PI3K-Akt pathway activation (activity score + 1.36, Spp1 FC = 3.65) and PPAR pathway inhibition (activity score −2.91, Fabp7 FC = 0.04). Collectively, these findings support a proposed model wherein Nano-Sb_2_O_3_ exposure is associated with PPAR inhibition and PI3K-Akt activation, correlating with SPP1 upregulation, inflammatory cascade activation, BTB-associated protein alterations, and male reproductive toxicity (Figure S5A–D; Table S3).

### 3.5. Spp1 Is a Prominently Upregulated Gene in Nano-Sb_2_O_3_-Induced Testicular Toxicity

**Figure 3:**
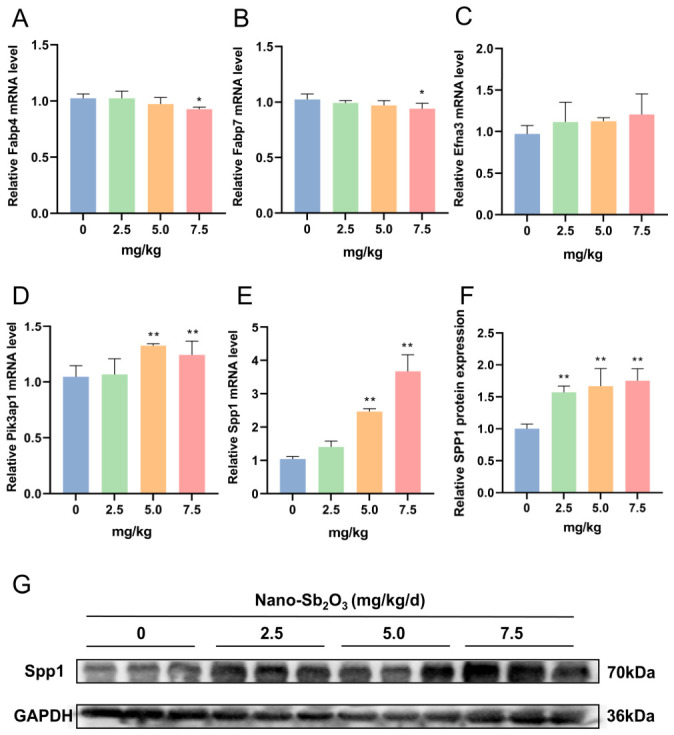
Validation of key gene expression in mouse testes after Nano-Sb_2_O_3_ exposure. (**A**–**E**) Relative mRNA levels of Fabp4, Fabp7, Efna3, Pik3ap1, and Spp1 in mouse testes (*n* = 3). (**F**,**G**) Relative protein expression of Spp1 and representative Western blot bands (GAPDH as internal control). Data are presented as mean ± SD (*n* = 5 per group). Figures (**A**–**F**): One-way ANOVA was applied to compare indicators among different groups. All ANOVA analyses were followed by Dunnett’s multiple comparisons test. * *p* < 0.05, ** *p* < 0.01 versus the control group. Graphs were generated using GraphPad Prism 9.0.

Based on transcriptome sequencing results, qPCR was used to verify the expression of genes involved in PPAR and PI3K-Akt pathways. Nano-Sb_2_O_3_ significantly downregulated Fabp4 and Fabp7 merely at high doses (Figure 3A,B), suggesting that PPAR-related lipid metabolism disorder was mainly triggered by high-dose exposure. No obvious expression alteration was found in Efna3 (Figure 3C), and Pik3ap1 exhibited only mild upregulation (Figure 3D).

In terms of expression variation, Pik3ap1 showed slight upregulation with fold change less than 2, without obvious expression alteration. In contrast, Spp1 was steadily upregulated in a dose-dependent manner at both mRNA and protein levels with consistent expression trends in vivo and in vitro (Figure 3E–G). Transcriptome analysis revealed that Spp1 exhibited the most prominent differential expression among the candidate genes, with a log_2_FC of 1.87 (Padj < 0.01) in the high-dose group. Consistent dose-dependent upregulation was further confirmed at both the mRNA and protein levels in vivo and in vitro (Figure 3E–G), consistent with its established roles in inflammatory activation, Sertoli cell function, and blood–testis barrier maintenance.

Supplementary analysis of SPP1 expression patterns provided additional correlative clues regarding its potential regulatory significance. Spearman correlation analysis, performed using transcriptomic data from all six samples (*n* = 6, exploratory), revealed that SPP1 expression tended to correlate inversely with PPAR-target genes (FABP4, CFD) and positively with the inflammatory mediator TNF and the PI3K adaptor PIK3AP1. STRING protein interaction network analysis suggested potential physical interactions between SPP1 and TNF-α (score = 0.715), IL-1β (score = 0.684), and N-cadherin/CDH2 (score = 0.657). Transcription factor enrichment analysis identified CEBPD and NR1H3/LXRα as potential upstream regulators of SPP1. Nevertheless, these analyses provide supportive clues for the potential involvement of SPP1 in linking pathway perturbations to inflammatory and BTB-related alterations (Figure S6). These findings suggest that Spp1 may be an important candidate contributor to Nano- Sb_2_O_3_-induced testicular toxicity.

### 3.6. Nano-Sb_2_O_3_ Suppresses TM4 Cell Viability and Migration, and Triggers Inflammatory Response

**Figure 4:**
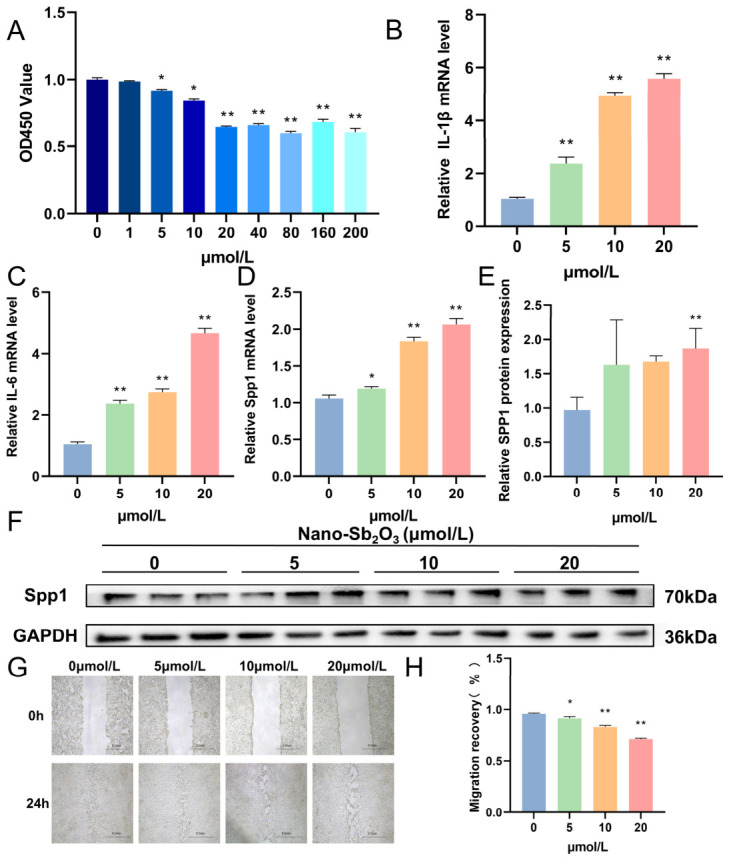
Effects of Nano-Sb_2_O_3_ exposure on TM4 cell viability, inflammation, migration, and Spp1 expression. (**A**) Cell viability assessed by CCK-8 assay (*n* = 6). (**B**,**C**) mRNA levels of pro-inflammatory cytokines IL-1β and IL-6 (*n* = 3). (**D**) Spp1 mRNA expression (*n* = 3). (**E**,**F**) Spp1 protein expression (quantification in E and representative Western blot bands in F; GAPDH as internal control, *n* = 3). (**G**,**H**) Wound-healing assay images (**G**) and quantitative analysis of migration area recovery (H, *n* = 3). Data are presented as mean ± SD (*n* = 5 per group). Figures (**A**–**E**): H: one-way ANOVA was applied to compare indicators among different groups. All ANOVA analyses were followed by Dunnett’s multiple comparisons test. * *p* < 0.05, ** *p* < 0.01 versus the control group. Graphs were generated using GraphPad Prism 9.0.

CCK-8 results showed that Nano-Sb_2_O_3_ did not affect TM4 cell activity at low a concentration, while it significantly inhibited cell viability in a dose-dependent manner above 5 μmol/L. At 5 μmol/L, the cell viability remained at 91.8 ± 0.9% of the control level; at 10 μmol/L, it decreased to 84.0 ± 1.9%; at 20 μmol/L, it further dropped to 64.7 ± 0.6%. We selected 0, 5, 10 and 20 μmol/L for follow-up experiments (Figure 4A). Wound healing assay suggested that Nano-Sb_2_O_3_ impaired TM4 cell migration. The cell migration rate gradually decreased with increasing concentration: compared with the control group, the migration rate was 91.6 ± 1.1% at 5 μmol/L, 83.0 ± 1.5% at 10 μmol/L, and 71.3 ± 0.9% at 20 μmol/L (Figure 4G,H).

To further validate the in vivo findings, we examined Spp1 expression in TM4 cells at both mRNA and protein levels using the selected concentrations. qPCR results showed Nano-Sb_2_O_3_ significantly upregulated Spp1 mRNA in a dose-dependent manner (Figure 4D). Western blot further confirmed the same rising trend of Spp1 protein expression. The changes in mRNA and protein levels were highly consistent (Figure 4E,F).

Given the upregulated Spp1 and its correlation with inflammation, we detected the expression of pro-inflammatory factors IL-6 and IL-1β. Their mRNA levels were distinctly increased in all treatment groups in a dose-dependent manner with statistically significant differences (Figure 4B,C). Specifically, IL-1β mRNA expression was increased by 2.4 ± 0.2-fold, 4.9 ± 0.1-fold, and 5.6 ± 0.2-fold at 5, 10, and 20 μmol/L (*p* < 0.01 vs. control). Similarly, IL-6 mRNA expression was upregulated by 2.4 ± 0.1-fold, 2.8 ± 0.1-fold, and 4.7 ± 0.1-fold at the corresponding concentrations (*p* < 0.01 vs. control).

### 3.7. Spp1 Knockdown Attenuates Cell Dysfunction and Inflammation and Partially Restores BTB-Associated Protein Expression

**Figure 5:**
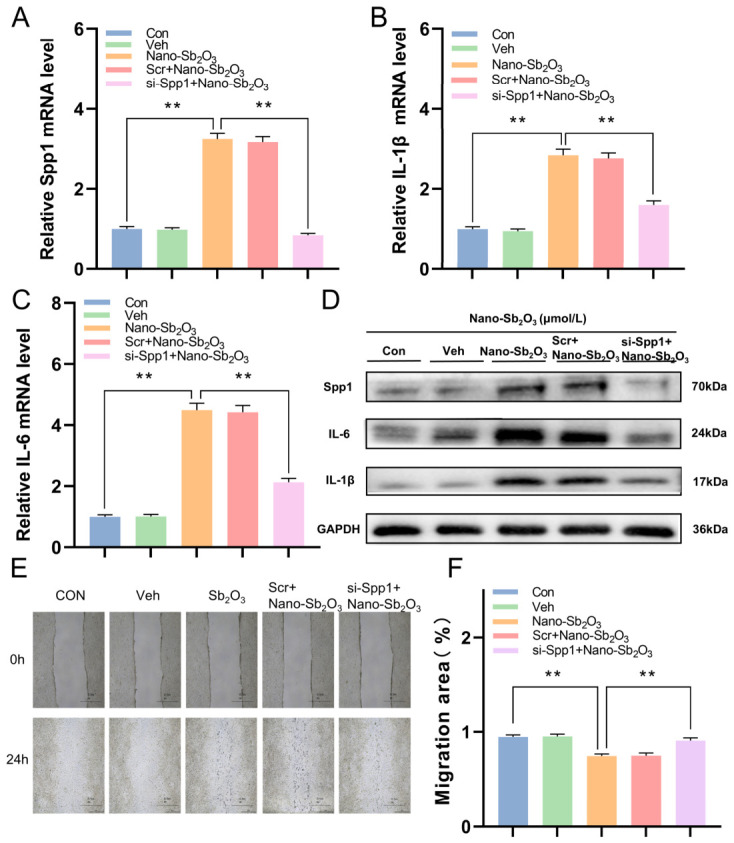
Knockdown of Spp1 alleviates Nano-Sb_2_O_3_-induced inflammation and migration inhibition in TM4 cells. (**A**–**C**) mRNA levels of Spp1, IL-1β, and IL-6 (*n* = 3). (**D**) Protein expression of Spp1, IL-6, and IL-1β (**E**,**F**) Wound-healing assay ((**E**): representative images; (**F**): quantitative analysis of migration area, *n* = 3). Data are presented as mean ± SD (*n* = 5 per group). Figures (**A**–**C**,**F**): one-way ANOVA was applied to compare indicators among different groups. All ANOVA analyses were followed by Dunnett’s multiple comparisons test. ** *p* < 0.01 versus the control group. Graphs were generated using GraphPad Prism 9.0.

qPCR results revealed that the mRNA levels of Spp1, IL-6 and IL-1β were significantly higher in Nano-Sb_2_O_3_ and Scr + Nano-Sb_2_O_3_ groups than those in control groups. Specifically, Spp1 mRNA expression was increased by 3.2 ± 0.2-fold in the Nano-Sb_2_O_3_ group, and the si-Spp1 transfection achieved a knockdown efficiency of 73.4 ± 2.1%, reducing Spp1 mRNA to 0.85 ± 0.05-fold relative to the control (Figure 5A). The expression of these genes was obviously decreased in si-Spp1 + Nano-Sb_2_O_3_ group (Figure 5B,C). Western blot results were consistent with qPCR data. The protein levels of Spp1, IL-6 and IL-1β were increased after Nano-Sb_2_O_3_ treatment, and declined after Spp1 interference (Figure 5D; Figure S7A–C).

The wound healing assay showed that cell migration was inhibited in Nano-Sb_2_O_3_ and Scr + Nano-Sb_2_O_3_ groups, while the migration ability was recovered in si-Spp1 + Nano-Sb_2_O_3_ group (Figure 5E,F).

**Figure 6:**
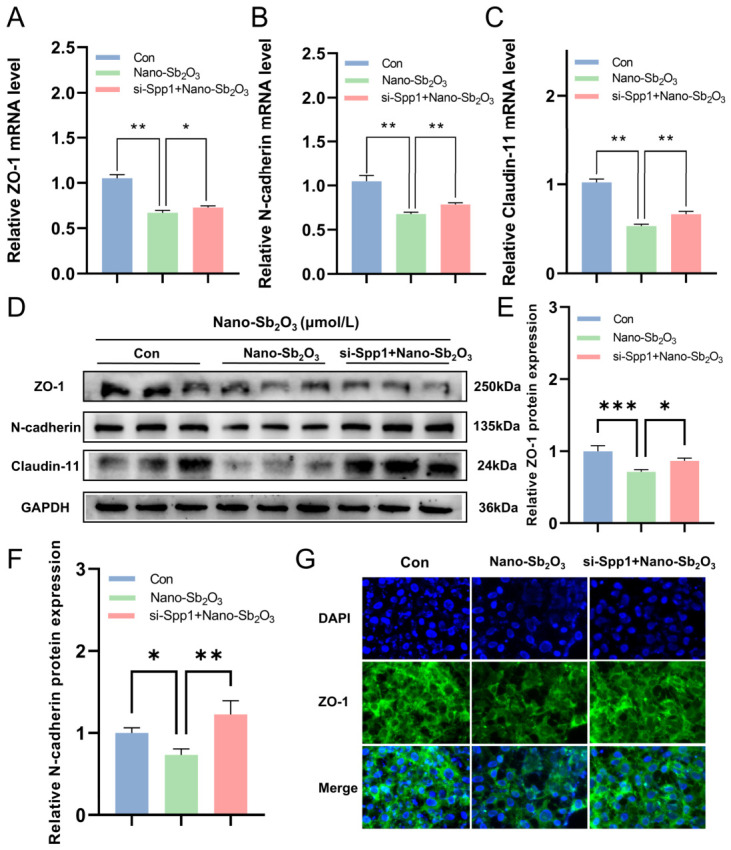
Knockdown of Spp1 reverses Nano-Sb_2_O_3_-induced downregulation of blood–testis barrier-related proteins in TM4 cells. (**A**–**C**) mRNA levels of ZO-1, Claudin-11, and N-cadherin (*n* = 3). (**D**–**G**) Protein expression of ZO-1, N-cadherin, and Claudin-11 ((**D**): representative Western blot bands; (**E**–**G**): quantitative analysis, GAPDH as internal control, *n* = 3). Data are presented as mean ± SD (*n* = 5 per group). Figures (**A**–**C**,**E**,**F**): one-way ANOVA was applied to compare indicators among different groups. All ANOVA analyses were followed by Dunnett’s multiple comparisons test. * *p* < 0.05, ** *p* < 0.01, and *** *p* < 0.001 versus the control group. Graphs were generated using GraphPad Prism 9.0.

qPCR results showed that Nano-Sb_2_O_3_ decreased the mRNA expression of ZO-1, Claudin-11 and N-cadherin in TM4 cells, to 66.2 ± 2.3%, 66.3 ± 3.1%, and 53.7 ± 2.8% of the control levels, respectively. The expression levels of these genes were increased after Spp1 knockdown: ZO-1 recovered to 72.5 ± 2.5%, Claudin-11 recovered to 77.8 ± 1.9%, and N-cadherin recovered by 78.6 ± 2.1% of the control levels (Figure 6A–C). Western blot analysis yielded consistent results. Nano-Sb_2_O_3_ reduced the protein levels of the three tight junction proteins, and Spp1 silencing increased their protein expression (Figure 6D–F and Figure S8A).

Immunofluorescence showed continuous and uniform distribution of ZO-1 at cell junctions in the control group. Nano-Sb_2_O_3_ reduced ZO-1 fluorescence intensity and disrupted its distribution. Higher fluorescence intensity and normal distribution of ZO-1 were observed in the si-Spp1 + Nano-Sb_2_O_3_ group (Figure 6G and Figure S8B). In addition, Annexin V/PI flow cytometry analysis showed no significant difference in the total apoptosis rate between Nano-Sb_2_O_3_-treated and control TM4 cells (*p* > 0.05, Figure S9), suggesting that apoptosis was not a major contributor under the present experimental conditions.

## 4. Discussion

Nano-Sb_2_O_3_, widely used as a synergistic flame retardant in industry, is continuously released into the environment during production, application, and disposal [33]. In the present study, we employed an integrated approach combining in vivo male mouse models and in vitro TM4 Sertoli cell experiments to investigate the mechanisms potentially involved in Nano-Sb_2_O_3_-induced reproductive injury, inflammatory activation, and alterations in blood–testis barrier (BTB)-associated proteins. We further examined the potential role of SPP1 in this pathological process, with the aim of providing mechanistic insights for health risk assessment of environmental Nano-Sb_2_O_33_ exposure.

Consistent with previous studies on nanomaterial-related male reproductive toxicity [34,35], our in vivo results demonstrated that Nano-Sb_2_O_3_ exposure caused dose-dependent impairment of male reproductive function and testicular histopathology, suggesting that Nano-Sb_2_O_3_ severely disturbs the spermatogenic microenvironment. Transcriptome screening identified the PPAR signaling pathway, PI3K-Akt signaling pathway, and the prominently upregulated gene Spp1, providing a foundation for mechanistic studies. Previous studies have demonstrated that antimony exposure induces oxidative stress, inflammatory responses, and cellular injury in multiple tissues [36,37]. Our results extend these observations by showing that Nano-Sb_2_O_3_ exposure also exerts significant adverse effects on the male reproductive system. The observed Sb accumulation in testicular tissues and elevated ROS levels suggest that oxidative stress may represent an early event contributing to Nano-Sb_2_O_3_-induced reproductive injury.

The hydrodynamic diameter of Nano-Sb_2_O_3_ measured in suspension (~297 nm) was substantially larger than its nominal primary particle size (50 nm), indicating particle aggregation under the experimental conditions. Such aggregation behavior may influence particle sedimentation, cellular uptake, tissue deposition, and biological responses. Similar discrepancies between primary particle size and hydrodynamic diameter have been reported for other metal oxide nanoparticles [38]. Therefore, the toxicological effects observed in the present study may reflect the biological behavior of aggregated Nano-Sb_2_O_3_ rather than individual primary nanoparticles.

Transcriptomic analysis revealed significant enrichment of the PPAR and PI3K-Akt signaling pathways following Nano-Sb_2_O_3_ exposure. In addition to conventional enrichment analyses, computational pathway perturbation approaches, including LINCS connectivity mapping, Enrichr pathway analysis, and PROGENy pathway activity inference, consistently supported suppression of PPAR signaling and activation of PI3K-Akt and inflammatory pathways. PPAR signaling is known to regulate lipid metabolism, energy homeostasis, and Sertoli cell function, whereas PI3K-Akt signaling plays important roles in cell survival, inflammatory regulation, and barrier maintenance [39,40]. Collectively, these findings provide complementary evidence supporting the potential involvement of the PPAR/PI3K-Akt signaling axis in Nano-Sb_2_O_3_-induced reproductive toxicity. Nevertheless, direct pharmacological or genetic perturbation studies of PPAR and PI3K-Akt pathway perturbations were not performed in the present study, which represents a limitation that warrants further validation in future investigations using specific pathway inhibitors or activators.

Among the identified differentially expressed genes, Spp1 emerged as one of the most significantly upregulated genes. SPP1 (osteopontin) is a multifunctional phosphoglycoprotein involved in immune regulation, inflammatory responses, cell adhesion, migration, and tissue remodeling [41]. Increasing evidence indicates that aberrant SPP1 expression contributes to chronic inflammation and tissue barrier dysfunction in multiple organs [42,43]. In the reproductive system, SPP1 has been reported to participate in cell–cell interactions and regulation of the testicular microenvironment [44]. Therefore, the marked upregulation of SPP1 observed in the present study suggested that it may participate in Nano-Sb_2_O_3_-induced inflammatory activation and alterations in BTB-associated proteins.

In vitro experiments further confirmed that Nano-Sb_2_O_3_ suppressed TM4 cell viability and migration, upregulated Spp1 expression, and activated inflammatory responses. Given the essential roles of Sertoli cells in supporting spermatogenesis and maintaining BTB integrity, the observed Nano-Sb_2_O_3_-induced Sertoli cell dysfunction suggests that inflammation-mediated impairment of Sertoli cells may represent a mechanism contributing to reproductive toxicity. Importantly, Spp1 knockdown attenuated inflammatory activation and partially restored BTB-associated protein expression. Given that the BTB is essential for maintaining the spermatogenic microenvironment [45], these findings suggest that SPP1-associated inflammatory responses may contribute to alterations in BTB-associated proteins and could participate in Nano-Sb_2_O_3_-induced male reproductive injury. Nevertheless, it should be noted that the current data support SPP1 as a contributing factor rather than a definitive mediator of BTB functional integrity. Consistently, Annexin V/PI flow cytometry confirmed no significant increase in apoptosis in TM4 cells following Nano-Sb_2_O_3_ exposure, further supporting that inflammatory activation, rather than apoptosis, represents the dominant cellular response under the present experimental conditions. Additionally, direct assessment of BTB permeability was not conducted in this study, and in vivo validation using Sertoli cell-specific Spp1-deficient models remains lacking. Future studies incorporating these approaches will be necessary to fully establish the functional role of SPP1 in BTB integrity. Furthermore, the sample size in the animal study was relatively small (*n* = 5 per group), which may limit statistical power. In addition, while intranasal instillation allows accurate dose administration, it does not fully mimic real-world occupational inhalation exposure [46,47].

## 5. Conclusions

In summary, this study indicates that Nano-Sb_2_O_3_ exposure induces dose-dependent male reproductive toxicity in mice and impairs the physiological function of TM4 Sertoli cells in vitro. Mechanistically, Nano-Sb_2_O_3_ upregulates SPP1 expression, which is associated with inflammatory activation and alterations in blood–testis barrier (BTB)-associated proteins. These findings identify SPP1 as a promising candidate molecule warranting further investigation in Nano-Sb_2_O_3_-induced male reproductive injury and provide experimental evidence for the risk assessment of Nano- Sb_2_O_3_ exposure. Nevertheless, given the correlative nature of the current evidence, direct causal validation through in vivo functional studies is needed to fully establish the role of SPP1 in BTB integrity.

## Data Availability

Data are contained within the article or Supplementary Material. All sequencing data were deposited in the GEO database with accession number GSE330989.

## Supplementary Materials

The following supporting information can be downloaded at: https://www.mdpi.com/article/10.3390/toxics14070569/s1, Table S1. Primary and secondary antibodies used in this study. Table S2. Primers used for quantitative real-time polymerase chain reaction (qRT-PCR). Table S3. Summary of computational pathway perturbation analysis. Figure S1: Characterization of Nano-Sb_2_O_3_. Figure S2. Antimony accumulation in testicular tissues following Nano-Sb_2_O_3_ exposure. Figure S3: Effects of Nano-Sb_2_O_3_ exposure on ROS levels in testicular tissues. Figure S4: Macroscopic observation and cross-sectional area analysis of mouse testes. Figure S5: Macroscopic observation and cross-sectional area analysis of mouse testes. Figure S6: Exploratory bioinformatics analysis of SPP1-associated pathways. Figure S7: Effects of Spp1 knockdown on the protein expression of Spp1, IL-6, and IL-1β in Nano-Sb_2_O_3_-treated TM4 cells. Figure S8: Effects of Spp1 knockdown on the expression of tight junction proteins in Nano-Sb_2_O_3_-treated TM4 cells. Figure S9: Flow cytometric analysis of apoptosis in TM4 cells after Nano-Sb_2_O_3_ treatment. Figure S10: Original uncropped Western blot images.
