# Supplementary material for: Spp1 Contributes to Nano-Antimony Trioxide-Induced Male Reproductive Toxicity Associated with Inflammatory Response and Blood–Testis Barrier-Related Alterations
Source: Toxics. 2026 Jun 28;14(7):569. doi: 10.3390/toxics14070569 (PMC13418332; doi:10.3390/toxics14070569)

Table S1. Primary and secondary antibodies used in this study.

Primary antibodies	Source	Catalog number
Spp1 Antibody	Proteintech	22952-1-AP (WB: 1:5000)
IL-6 Antibody	Affinity	DF6087 (WB: 1:1000)
IL-1β Antibody	Affinity	AF4006 (WB: 1:1000)
N-Cadherin polyclonal antibody	Bioworld	BS80427 (WB: 1:500)
ZO-1 polyclonal antibody	Bioworld	BS71522 (WB: 1:500)
Claudin-11 (S198) polyclonal antibody	Bioworld	BS1065 (WB: 1:500)
ZO-1 Polyclonal antibody	Proteintech	21773-1-AP(IF: 1:400)
Rabbit polyclonal anti-GAPDH	Affinity	AF7021 (WB: 1:5000)

Table S2. Primers used for quantitative real-time polymerase chain reaction (qRT-PCR).

Gene	Forward Primer (5'→3')	Reverse Primer (5'→3')	Product Size (bp)
Gapdh	AGGTCGGTGTGAACGGATTTG	GGGGTCGTTGATGGCAACA	121
Pik3ap1	CTACAGCCCAGATGCGGAG	AGCCGGTATGTCTGCGTCT	134
Fabp7	GGACACAATGCACATTCAAGAAC	CCGAACCACAGACTTACAGTTT	127
Fabp4	AAGGTGAAGAGCATCATAACCCT	TCACGCCTTTCATAACACATTCC	131
SPP1	ATCTCACCATTCGGATGAGTCT	TGTAGGGACGATTGGAGTGAAA	118
Efna3	AGGTGAACGTGAACGACTATCT	GCGCTGTAACGCTGGAACT	106
ZO-1	GCCGCTAAGAGCACAGCAA	TCCCCACTCTGAAAATGAGGA	101
Claudin-11	GCTGCTGGCCTTCATCGTAG	GGTAGTCCTTGTCGTGCTTCTG	123
N-Cadherin	TTGAAGATGATGATGAAGGTGAA	CTTGAAGTCGATTGTTGATGAA	115
IL-6	GAGGATACCACTCCCAACAGACC	AAGTGCATCATCGTTGTTCATACA	141
IL-1 β	GCTGAAAGCTCTCCACCTCA	AGGCCACAGGTATTTTGTCG	117

Table S3. Summary of computational pathway perturbation analysis.

Analysis Category	Database	Pathway / Term	P / Score	Z-score	Core DEGs
Pathway Enrichment	KEGG 2021	Linoleic acid metabolism	0.00238	30.75	-
Pathway Enrichment	KEGG 2021	Steroid hormone biosynthesis	0.01020	14.05	-
Pathway Enrichment	KEGG 2021	Arachidonic acid metabolism	0.01020	14.05	-
Pathway Enrichment	KEGG 2021	PPAR signaling pathway	0.01475	11.50	FABP4, FABP7
Pathway Enrichment	KEGG 2021	Metabolism by CYP450	0.01552	11.19	
Pathway Enrichment	KEGG 2021	ECM-receptor interaction	0.02045	9.62	SV2C, SPP1
Pathway Enrichment	KEGG 2021	Inflamm. mediator TRP	0.02499	8.62	PLA2G4B, TRPV1
Pathway Enrichment	KEGG 2021	Drug metabolism	0.02991	7.80	
Pathway Enrichment	KEGG 2021	Serotonergic synapse	0.03250	7.45	
Pathway Enrichment	KEGG 2021	PI3K-Akt signaling	0.05857	3.56	EFNA3, SPP1, PIK3AP1
Pathway Enrichment	WikiPathways 2021	TYROBP causal network	0.01020	14.05	PLEK, SPP1
Pathway Enrichment	WikiPathways 2021	PPAR signaling	0.01221	12.75	FABP4, FABP7
Pathway Enrichment	WikiPathways 2021	Osteopontin Signaling	0.03203	33.91	SPP1
Hallmark	MSigDB 2020	TNF-alpha via NF-kB	0.01368	6.40	CEBPB, CCRL2, PLEK
Hallmark	MSigDB 2020	Inflammatory Response	0.01368	6.40	TPBG, CCRL2, NMUR1
Hallmark	MSigDB 2020	Interferon Alpha Response	0.02452	8.71	CCRL2, IFIT3
Connectivity	L1000CDS2	Reverse (antagonist)	-	-	L-690,488 (score=0.161)
Connectivity	L1000CDS2	Agonist mode	-	-	-666 (score=0.167)

Pathway Activity	PROGENy	PI3K-Akt [ACTIVATED]	+1.36	-	Spp1, Efna3, Pik3ap1
Pathway Activity	PROGENy	PPAR [INHIBITED]	-2.91	-	Fabp7, Fabp4, Cfd
Pathway Activity	PROGENy	NF-kB/Inflamm. [ACTIVATED]	+1.55	-	Cebpb, Ccrl2, Trpv1

Figure S1. Characterization of nano-antimony trioxide (Nano-Sb_2O_3). (a) Scanning electron microscopy (SEM) image showing the morphology of Sb_2O_3 nanoparticles. Scale bar: 500 nm. (b) Dynamic light scattering (DLS) analysis showing the hydrodynamic particle size distribution of Sb_2O_3 in normal saline, with an average diameter of 297 nm. (c) Zeta potential distribution of Sb_2O_3 in normal saline, showing an average value of -13.6 ± 0.61 mV.

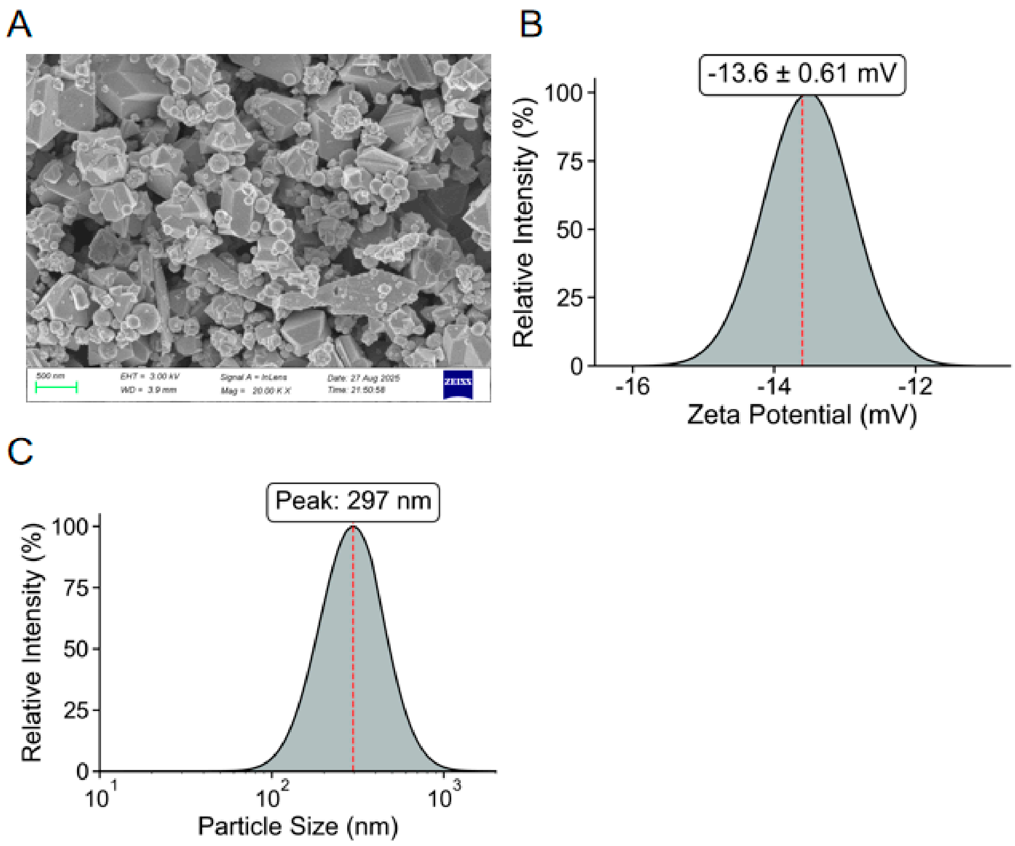

Figure S2. Sb concentrations in testicular tissues were determined by ICP-OES. Data are

presented as mean ± SD (n = 5 per group). Statistical analysis was performed using one-way analysis of variance (ANOVA) followed by Dunnett's multiple comparisons test. *P < 0.05, **P < 0.01, and ***P < 0.001 versus the control group.

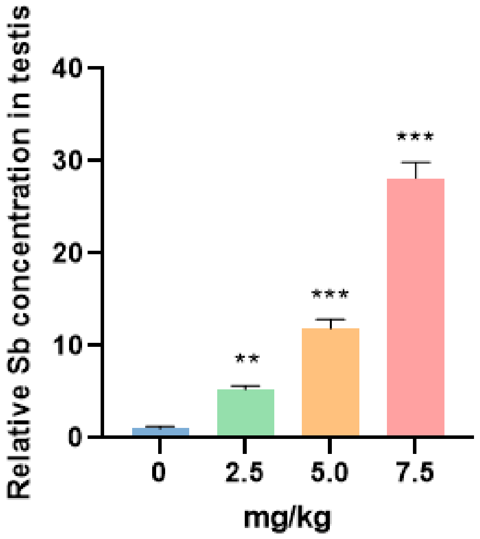

Figure S3. Relative ROS levels in testicular tissues. Data are presented as mean ± SD (n = 5 per group). Statistical analysis was performed using one-way analysis of variance (ANOVA) followed by Dunnett's multiple comparisons test. *P < 0.05, **P < 0.01, and ***P < 0.001 versus the control group.

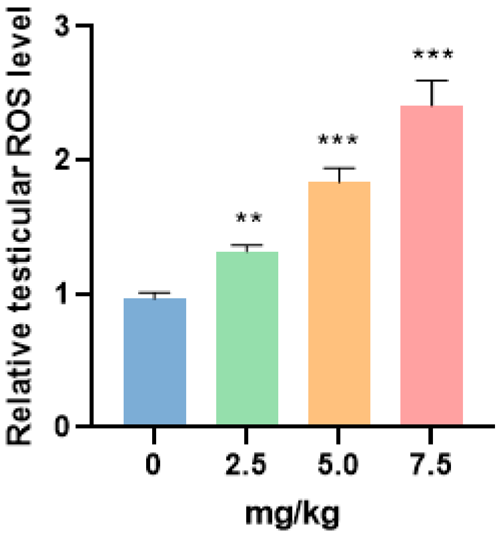

Figure S4. Effects of Nano-Sb_2O_3 exposure on gross testicular morphology. (A)

Representative photographs of testes from mice exposed to Nano-Sb_2O_3 (0, 2.5, 5.0, and 7.5 mg/kg/day). (B) Quantification of testicular area. Data are presented as mean ± SD (n = 5 per group). Statistical analysis was performed using one-way analysis of variance (ANOVA) followed by Dunnett's multiple comparisons test. No significant changes in testicular area were observed following Nano-Sb_2O_3 exposure.

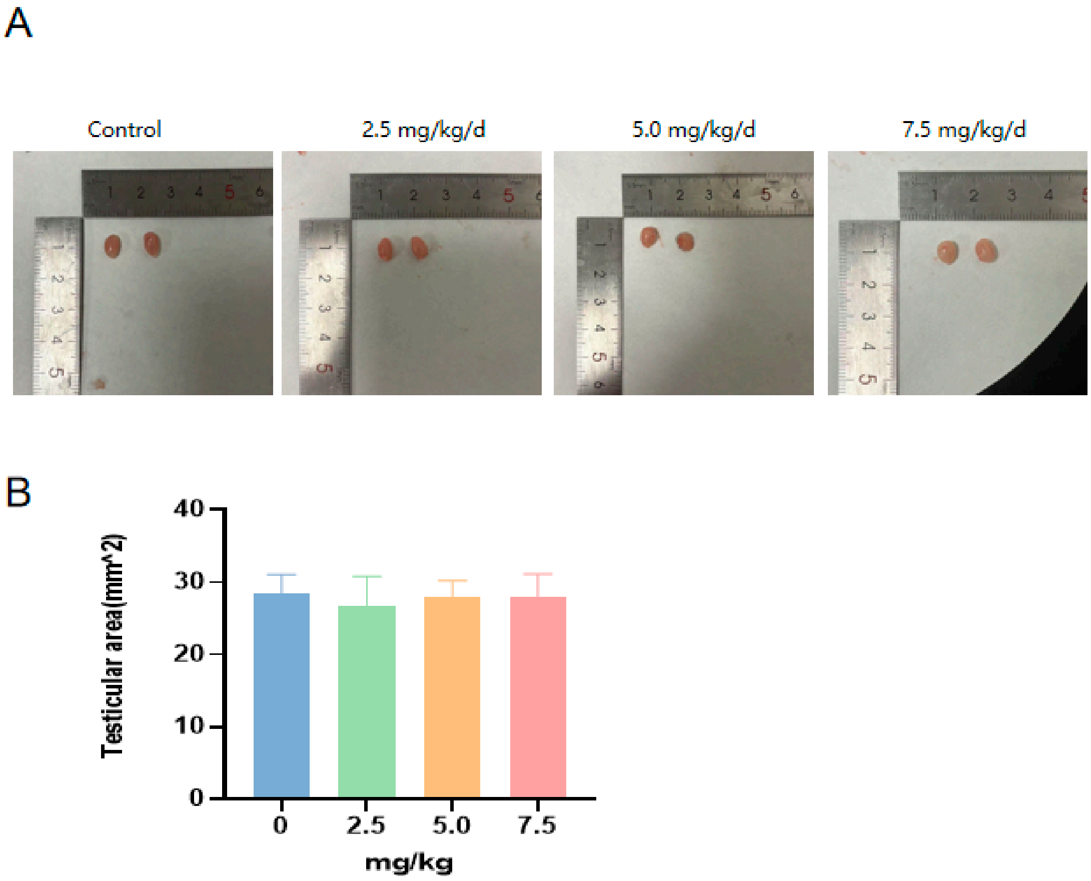

Figure S5. Virtual pathway perturbation analysis. (A) LINCS L1000CDS[2] connectivity mapping. Bar chart showing the top 10 small molecules producing gene expression signatures that reverse the Nano-Sb_2O_3 DEG profile, color-coded by mechanism of action. (B) KEGG pathway enrichment analysis. Horizontal bar chart of the top 10 enriched pathways ($-\log_{10}P$). The PPAR signaling pathway is highlighted in red, and the PI3K-Akt pathway is highlighted in pink. The dashed line indicates the P=0.05 significance threshold. (C) Pathway perturbation signatures. Left panel: MSigDB Hallmark enrichment analysis ($-\log_{10}P$) showing significant enrichment of TNF-α/NF-κB signaling, Inflammatory Response, and Interferon Alpha Response (dashed line: P=0.05). Right panel: PROGENy pathway activity scores showing the mean $\log_2FC$ of core DEGs

per pathway, with positive values (red) indicating activation and negative values (blue) indicating inhibition. (D) Key DEG expression levels. Grouped bar chart showing TPM values for core genes in PPAR (Fabp7, Fabp4, Cfd), PI3K-Akt (Spp1, Efna3, Pik3ap1), Inflammatory (Cebpb, Ccrl2, Trpv1), and Metabolic (Aldh3a1, Cyp2e1) pathways in treatment (red) versus control (blue) groups. *P<0.05.

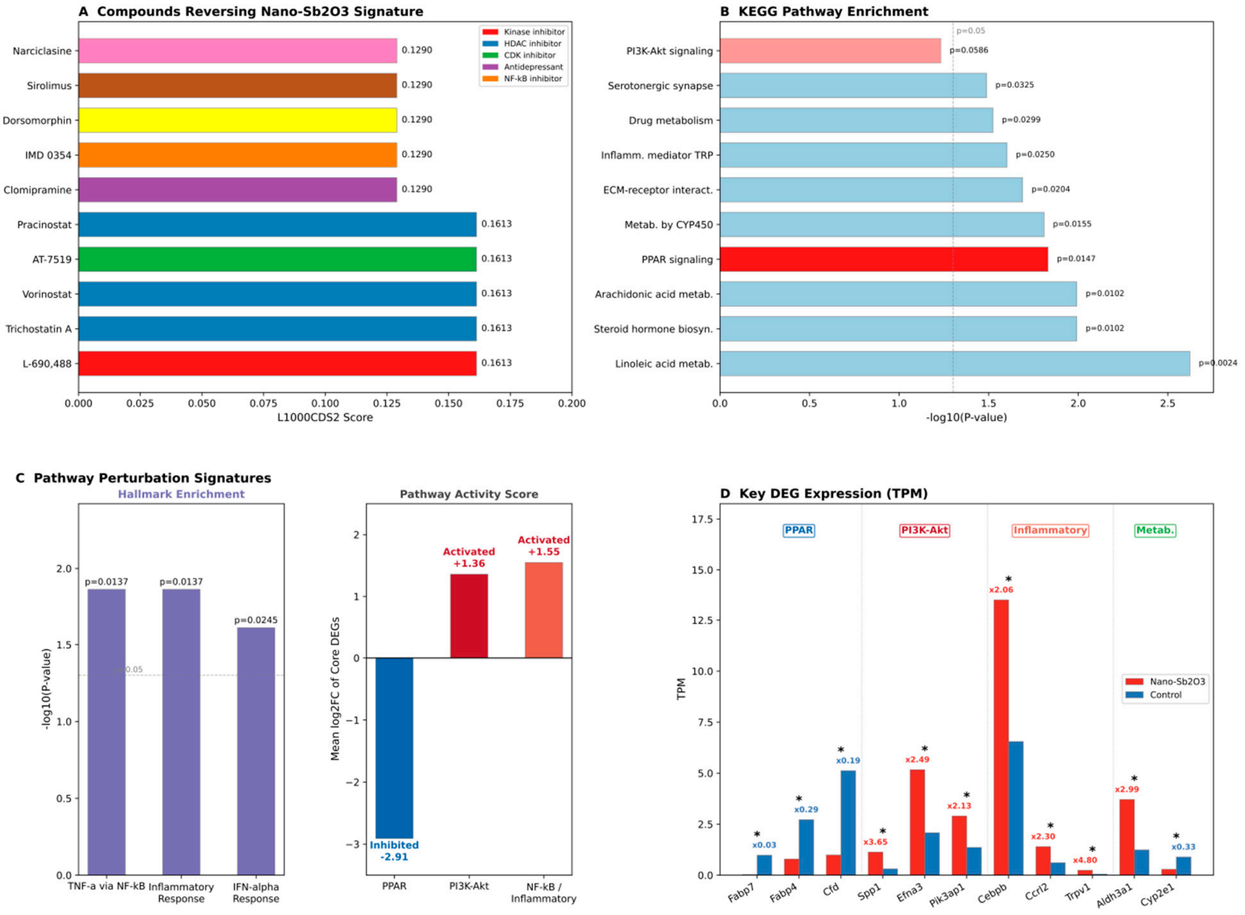

Figure S6. Exploratory bioinformatics analysis of SPP1-associated pathways. (A) Spearman correlation between SPP1 and selected genes (n = 6; 3 treated + 3 control), grouped by pathway (orange, inflammatory; blue, PPAR targets; purple, PI3K-Akt; green, BTB). (B) Upstream transcription factors of SPP1 predicted by Enrichr (CEBPD and NR1H3/LXRα highlighted). (C) STRING-predicted protein interaction network of SPP1 (medium confidence ≥ 0.400). All analyses in this figure are exploratory and hypothesis-generating; due to the limited sample size (n = 6), these findings do not establish causality.

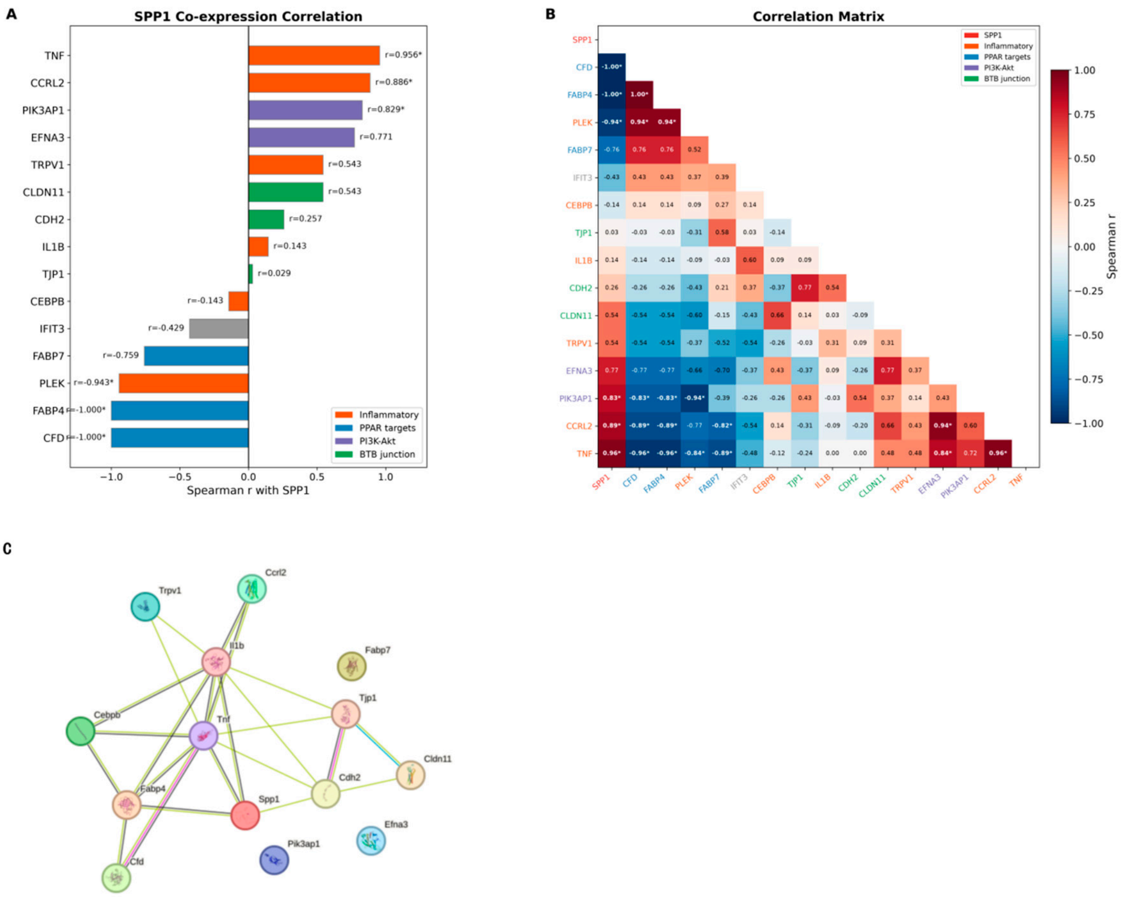

Figure S7. Effects of Nano-Sb_2O_3 exposure and Spp1 knockdown on the protein expression of Spp1, IL-6, and IL-1β in TM4 cells. (A-C) Quantitative analysis of the relative protein expression levels of Spp1 (A), IL-6 (B), and IL-1β (C) in different groups (CON, Veh, Nano-Sb_2O_3, Scr+Nano-Sb_2O_3, si-Spp1+Nano-Sb_2O_3). Data are presented as mean ± SD (n = 5 per group). Statistical analysis was performed using one-way analysis of variance (ANOVA) followed by Dunnett's multiple comparisons test. *P < 0.05, **P < 0.01, and ***P < 0.001 versus the control group.

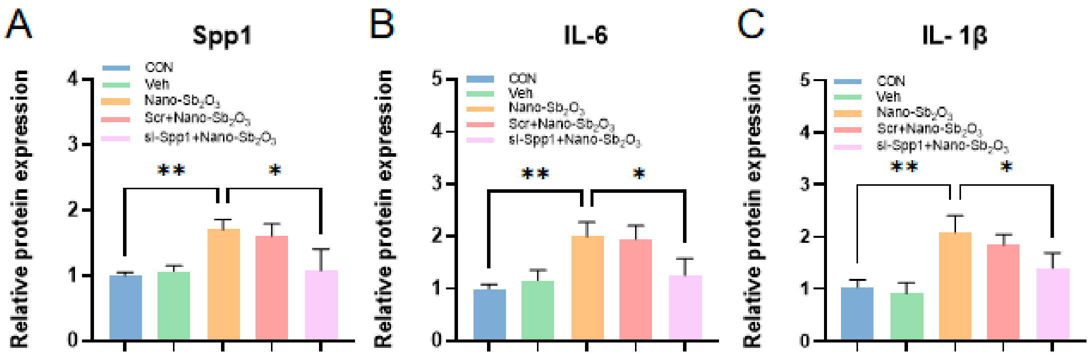

Figure S8. Effects of Nano-Sb_2O_3 exposure and Spp1 knockdown on the expression of tight junction proteins in TM4 cells. (A) Relative protein expression of N-cadherin. (B) Relative fluorescence intensity of ZO-1. Groups: control (Con), Nano-Sb_2O_3 exposure (Nano-Sb_2O_3), and Spp1 knockdown plus Nano-Sb_2O_3 exposure (si-Spp1 + Nano-Sb_2O_3). Data are presented as mean ± SD (n = 5 per group). Statistical analysis was performed using one-way analysis of variance (ANOVA) followed by Dunnett's multiple comparisons test. *P < 0.05, **P < 0.01, and ***P < 0.001 versus the control group.

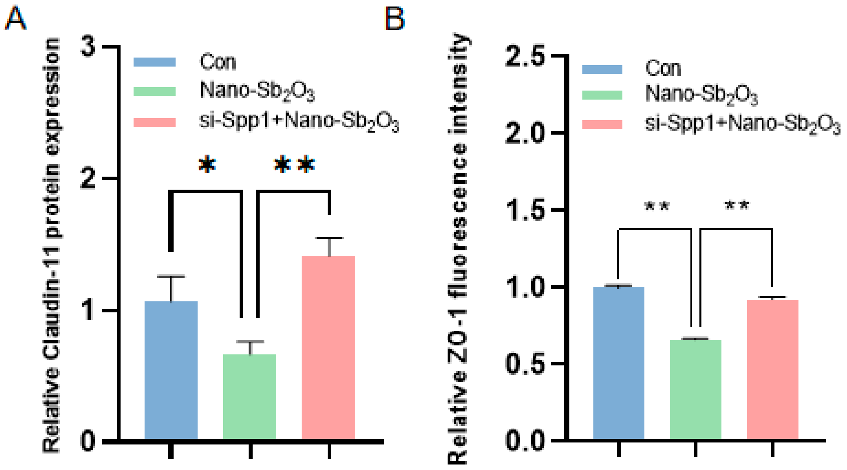

Figure S9 :Flow cytometric analysis of apoptosis in TM4 cells after Nano-Sb_2O_3 treatment. (A) Representative flow cytometry dot plots of TM4 cell apoptosis. Q3: viable cells; Q4: early apoptosis; Q2: late apoptosis; Q1: necrotic cells. (B): Quantification of total apoptosis rate (Q4 + Q2) in TM4 cells. Data are mean ± SD (n = 3). Data are presented as mean ± SD (n = 5 per group). Statistical analysis was performed using one-way analysis of variance (ANOVA) followed by Dunnett's multiple comparisons test. ns: not significant.

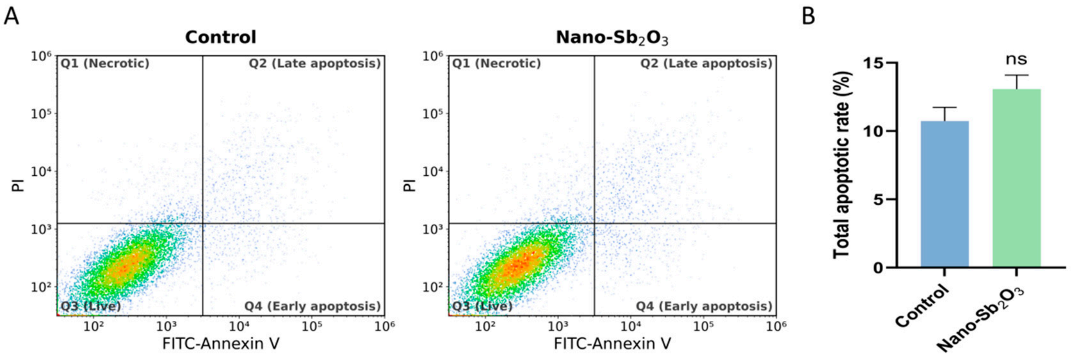

Figure S10 : Original uncropped Western blot images .

(1) Figure 3G Spp1 Marker

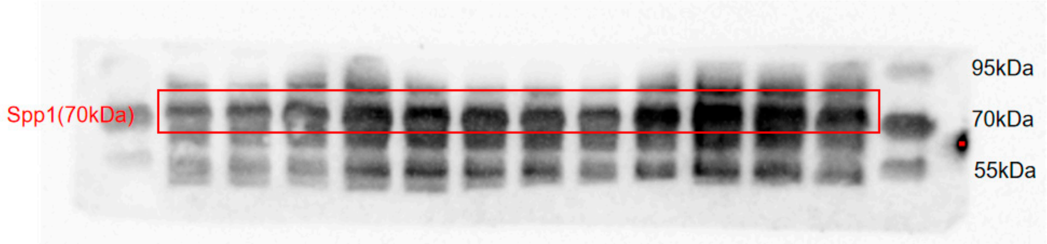

(2)Figure 3G GAPDH Marker

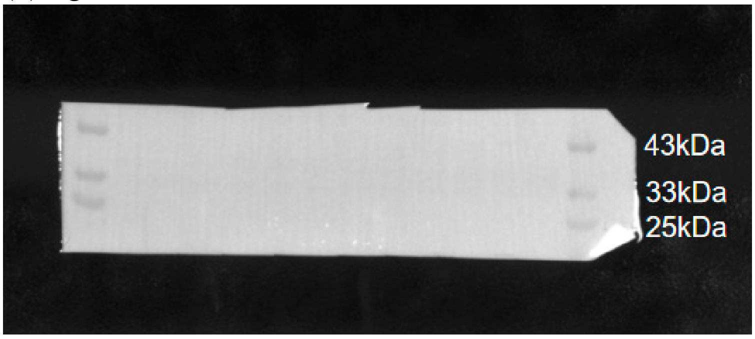

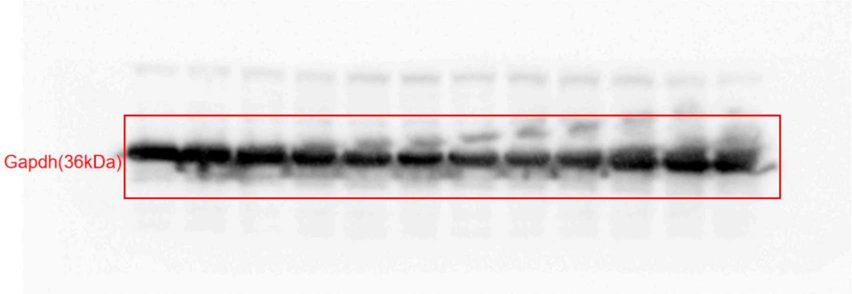

(3) Figure 4F Spp1 Marker

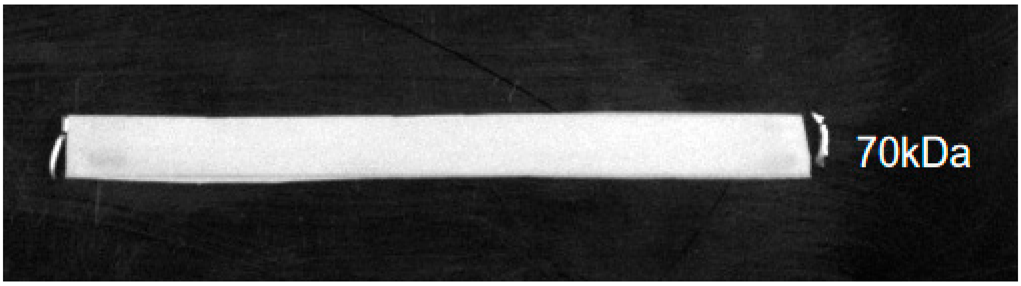

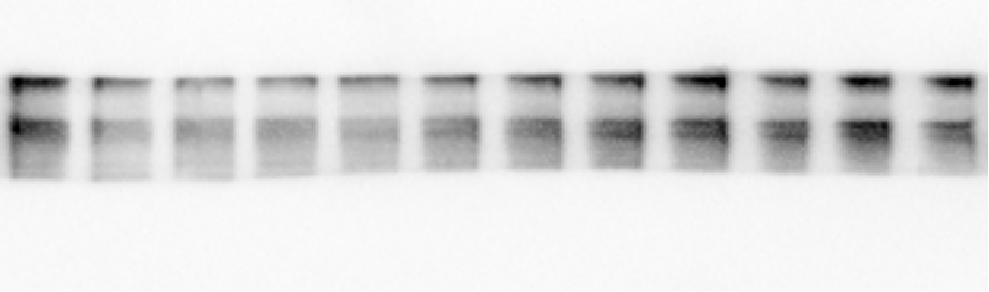

(4) Figure 4F GAPDH Marker

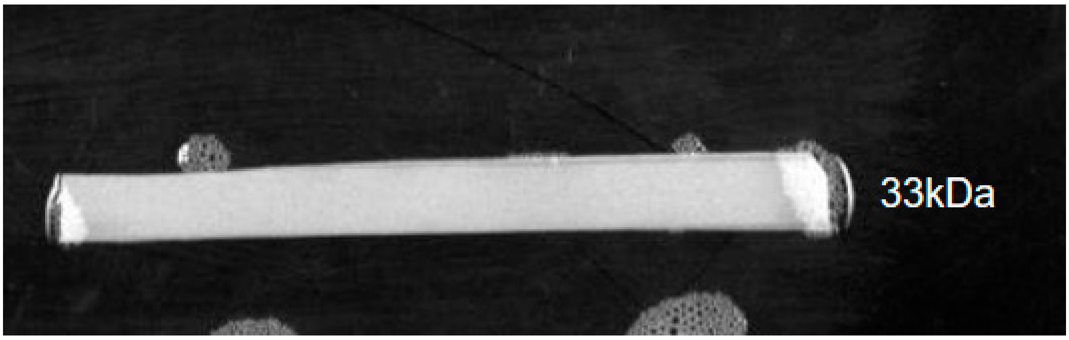

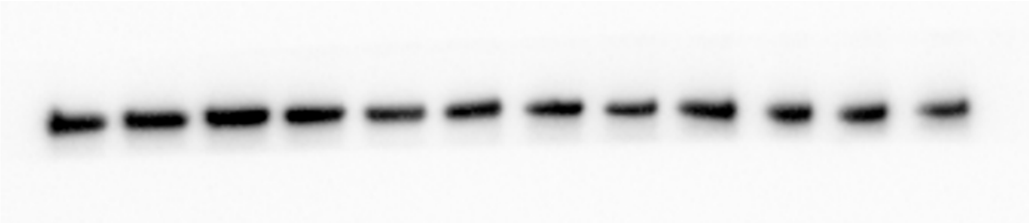

(5) Figure 5D Spp1 Marker

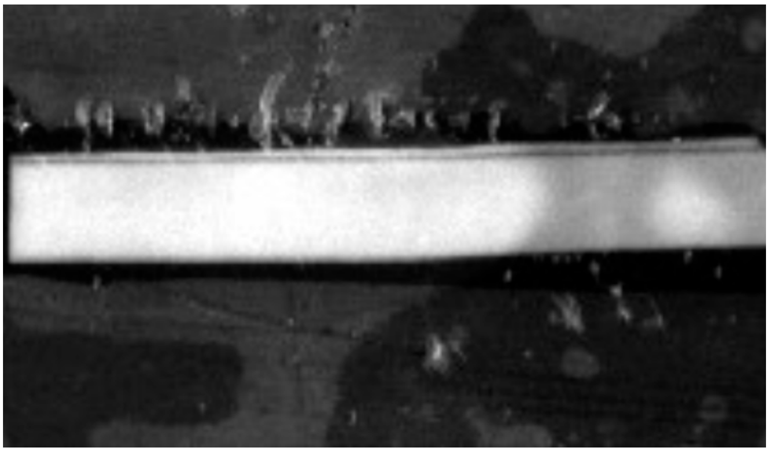

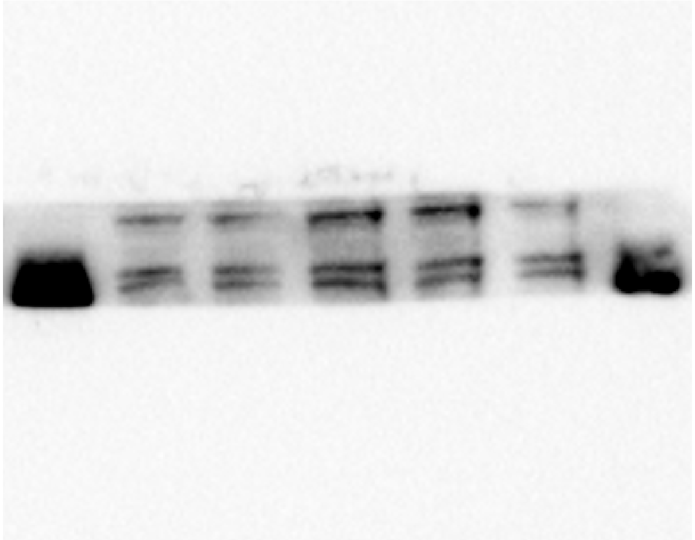

(6) Figure 5D IL-6 Marker

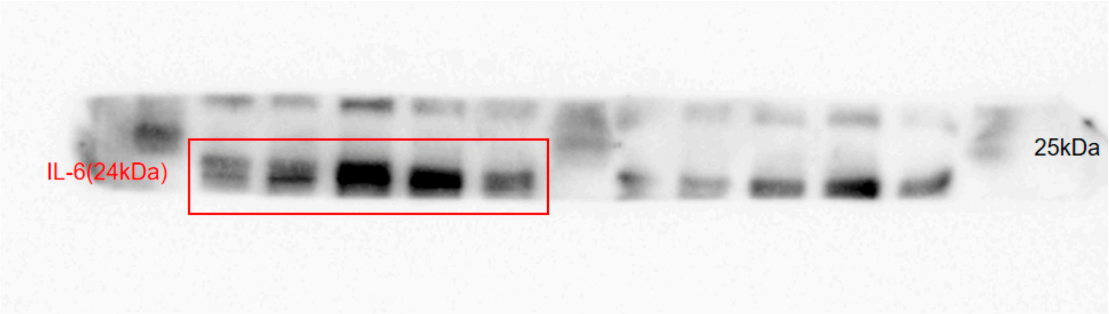

(7) Figure 5D IL-1β Marker

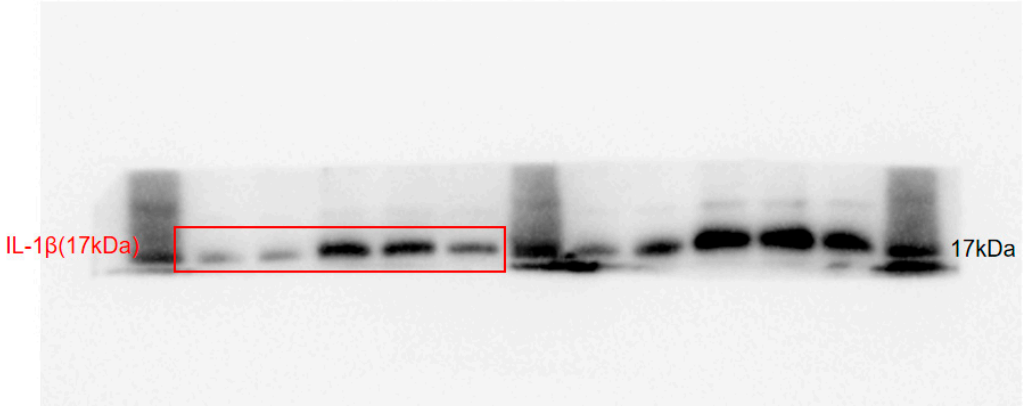

(8) Figure 5D GAPDH Marker

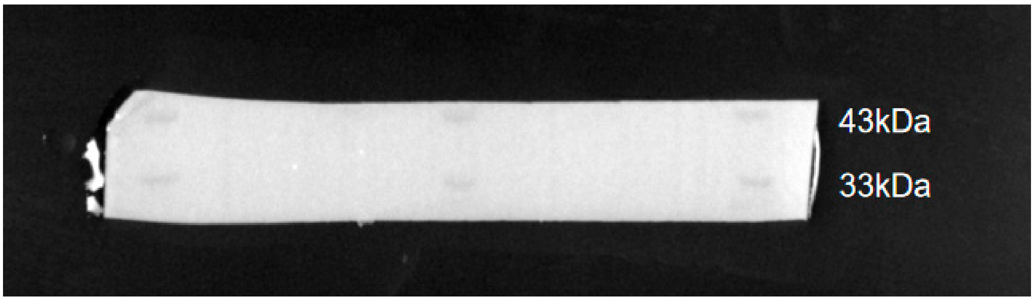

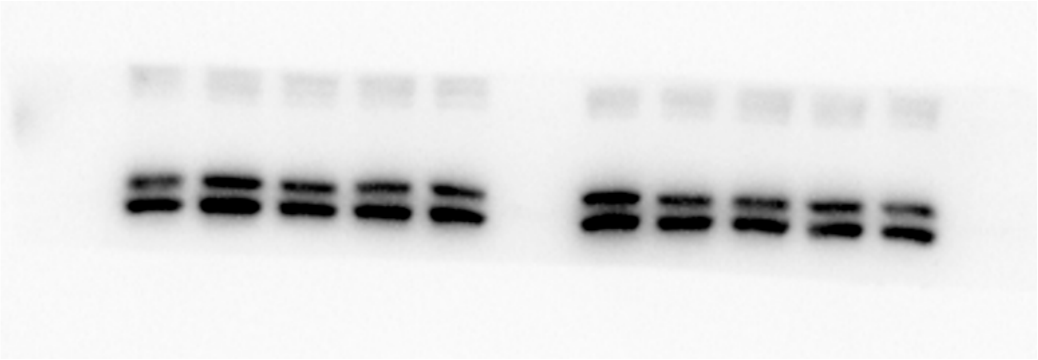

(9) Figure 6D ZO-1 Marker

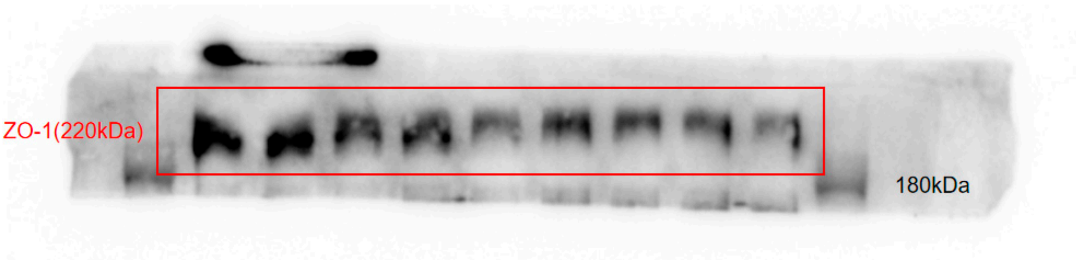

(10)Figure 6D N-Cadherin Marker

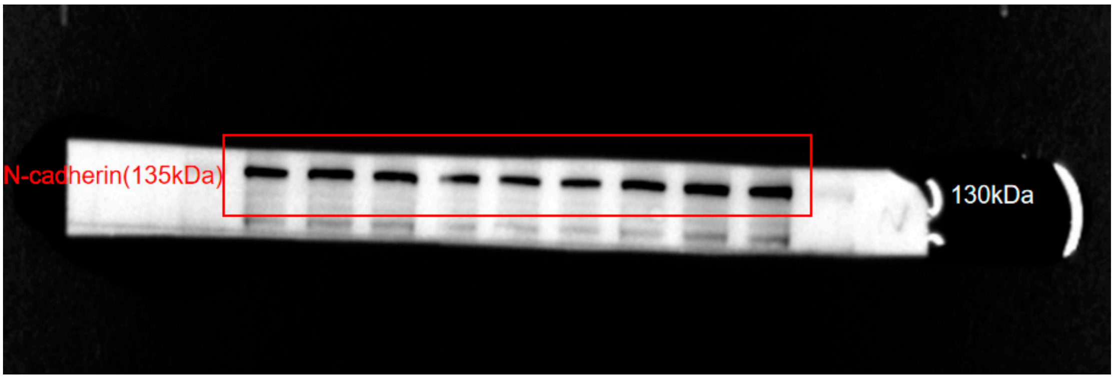

(11)Figure 6D Claudin-11 Marker

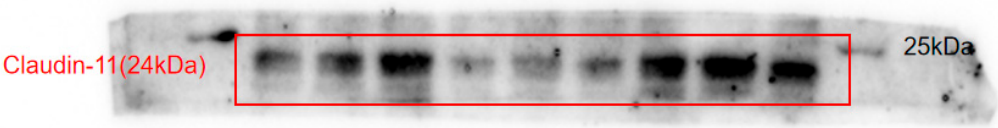

(12)Figure 6D GAPDH Marker

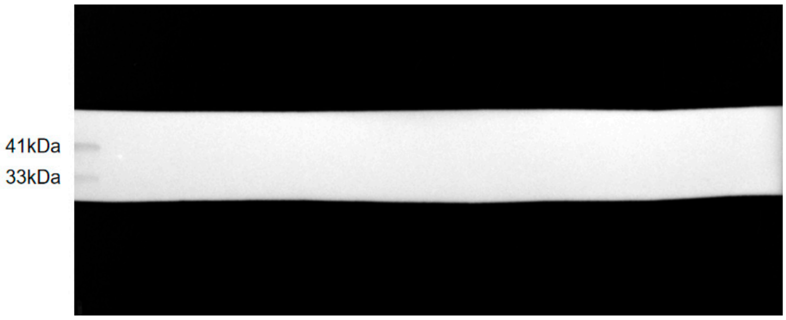

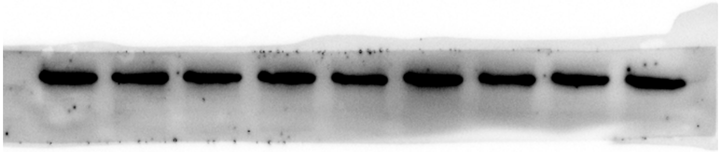

Supplement: Supplementary file 1 [file toxics-14-00569-s001.zip › toxics-4354996-supplementary.pdf]
